# Biophysical classification of a *CACNA1D* de novo mutation as a high-risk mutation for a severe neurodevelopmental disorder

**DOI:** 10.1186/s13229-019-0310-4

**Published:** 2020-01-08

**Authors:** Nadja T. Hofer, Petronel Tuluc, Nadine J. Ortner, Yuliia V. Nikonishyna, Monica L. Fernándes-Quintero, Klaus R. Liedl, Bernhard E. Flucher, Helen Cox, Jörg Striessnig

**Affiliations:** 1 Department of Pharmacology and Toxicology, Centre for Molecular Biosciences, University of Innsbruck, Innrain 80/82, 6020 Innsbruck, Austria; 2 Institute of General, Inorganic and Theoretical Chemistry, Centre for Molecular Biosciences, University of Innsbruck, Innsbruck, Austria; 3 Division of Physiology, Department of Physiology and Medical Physics, Medical University Innsbruck, 6020 Innsbruck, Austria; 4 West Midlands Regional Clinical Genetics Service, Birmingham Women’s and Children’s Hospital, National Health Service Foundation Trust, B15 2TG, Birmingham, UK

**Keywords:** Autism spectrum disorder, Neurodevelopmental disorder, *CACNA1D*, Gain-of-function mutation, L-type Ca^2+^-channels

## Abstract

**Background:**

There is increasing evidence that de novo *CACNA1D* missense mutations inducing increased Cav1.3 L-type Ca^2+^-channel-function confer a high risk for neurodevelopmental disorders (autism spectrum disorder with and without neurological and endocrine symptoms). Electrophysiological studies demonstrating the presence or absence of typical gain-of-function gating changes could therefore serve as a tool to distinguish likely disease-causing from non-pathogenic de novo *CACNA1D* variants in affected individuals. We tested this hypothesis for mutation S652L, which has previously been reported in twins with a severe neurodevelopmental disorder in the Deciphering Developmental Disorder Study, but has not been classified as a novel disease mutation.

**Methods:**

For functional characterization, wild-type and mutant Cav1.3 channel complexes were expressed in tsA-201 cells and tested for typical gain-of-function gating changes using the whole-cell patch-clamp technique.

**Results:**

Mutation S652L significantly shifted the voltage-dependence of activation and steady-state inactivation to more negative potentials (~ 13–17 mV) and increased window currents at subthreshold voltages. Moreover, it slowed tail currents and increased Ca^2+^-levels during action potential-like stimulations, characteristic for gain-of-function changes. To provide evidence that only gain-of-function variants confer high disease risk, we also studied missense variant S652W reported in apparently healthy individuals. S652W shifted activation and inactivation to more positive voltages, compatible with a loss-of-function phenotype. Mutation S652L increased the sensitivity of Cav1.3 for inhibition by the dihydropyridine L-type Ca^2+^-channel blocker isradipine by 3–4-fold.

Conclusions and limitations

Our data provide evidence that gain-of-function *CACNA1D* mutations, such as S652L, but not loss-of-function mutations, such as S652W, cause high risk for neurodevelopmental disorders including autism. This adds *CACNA1D* to the list of novel disease genes identified in the Deciphering Developmental Disorder Study. Although our study does not provide insight into the cellular mechanisms of pathological Cav1.3 signaling in neurons, we provide a unifying mechanism of gain-of-function *CACNA1D* mutations as a predictor for disease risk, which may allow the establishment of a more reliable diagnosis of affected individuals. Moreover, the increased sensitivity of S652L to isradipine encourages a therapeutic trial in the two affected individuals. This can address the important question to which extent symptoms are responsive to therapy with Ca^2+^-channel blockers.

## Background

In electrically excitable cells, Ca^2+^ inward current through voltage-gated Ca^2+^-channels (VGCCs; Cav) contributes to membrane depolarization and plays a key role in coupling electrical activity to intracellular Ca^2+^-dependent signaling processes (for review, see [1–3]). Therefore, VGCCs control essential physiological processes, such as hormone secretion, muscle contraction, sinoatrial node pacemaking, and sensory function. In the brain, they trigger neurotransmitter release, shape neuronal excitability, and couple excitation to gene expression associated with synaptic plasticity and different types of learning and memory [1, 3–5]. VGCC activity is fine-tuned to the specific requirements of cellular functions. This is accomplished by the functional heterogeneity and diverse subcellular targeting of ten pore-forming α_1_-subunit isoforms [6], several accessory β- and α_2_δ-subunits as well as by alternative splicing and post-translational modification (for a review, see [3]).

L-type Ca^2+^ channels (LTCCs; Cav1) form one of the three main families of VGCCs. From the four members (Cav1.1–Cav1.4), Cav1.2 and Cav1.3 are expressed in most electrically excitable cells, including a wide variety of brain regions [3]. Both are located post-synaptically at somatodendritic locations and serve a key role in activity-dependent gene transcription [1, 3, 5]. Genetic variants in both channels have been associated with neuropsychiatric disease risk. Multiple common intronic single nucleotide polymorphisms (SNPs) in Cav1.2 (*CACNA1C gene*) LTCCs have consistently been associated with bipolar disorder and schizophrenia, although the consequences of these polymorphisms for Cav1.2 function remain unknown (for review, see [7–9]). Very rare de novo *CACNA1C* missense mutations cause Timothy Syndrome, a severe disease with lethal arrhythmias, facial dysmorphism, syndactyly and autism spectrum disorder (ASD) in surviving patients [10–12]. Together these findings have triggered new interest in clinical trials to repurpose LTCC blockers (“Ca^2+^-antagonists”), licensed as antihypertensive drugs since decades, also for the treatment of mood disorders [13].

We [14–16] and others [17–20] have recently provided accumulating evidence that de novo missense mutations in the pore-forming α_1_-subunit of Cav1.3 LTCCs (*CACNA1D*) confer high risk for neurodevelopmental disorders in humans. Symptoms range from ASD with (mutations A749G, Q547H [14, 20];) and without (G407R [14]) intellectual disability to more severely affected patients with seizures, muscle hypotonia, and global developmental delay (V401L [15]). Some patients also exhibit additional endocrine symptoms (primary aldosteronism or hyperinsulinism; G403D, I750M [17, 18]) due to the expression of Cav1.3 in adrenal zona glomerulosa cells and pancreatic β-cells (for a review, see [3]). Our studies revealed that all these genetic variants are not present in healthy parents or unaffected siblings and are absent in 141,456 reference genomes of controls without pediatric disease (gnomAD database, [21]). This is consistent with high penetrance and strongly supports a likely causative role of these mutations. Moreover, electrophysiological analysis of six mutations (from seven of these patients) after expression in HEK-293 cells revealed a highly consistent pattern of functional changes: they all induce gating changes that can enhance Cav1.3 Ca^2+^-current through these channels, in particular at subthreshold voltages. This gain-of-function is evident from a drastic slowing of channel inactivation and/or by facilitation of channel opening at more negative voltages [16]. Therefore, the demonstration of such typical gain-of-function gating changes in functional studies may allow to distinguish likely pathogenic from non-pathogenic *CACNA1D* missense variants and help in the genetic diagnosis of individuals with neurodevelopmental disorders. This appears necessary because several genetic studies failed to classify *CACNA1D* missense variants as high-risk mutations and *CACNA1D* as a high-risk gene for neurodevelopmental disorders, including ASD [14, 15, 22, 23]. For example, gain-of-function *CACNA1D* mutation G407R in a patient with ASD has been identified, but has not been classified as high-risk mutation. However, functional analysis revealed typical gain-of-function changes, which strongly support its pathogenic potential [14].

In contrast to de novo gene-disrupting mutations (nonsense, splice site, frameshift), which cause a protein loss-of-function, the prediction of the pathogenic potential of missense variants is more difficult because in most cases their functional consequences cannot be predicted by bioinformatics tools. While our data argue for a high disease risk due to Cav1.3 gain-of-function, heterozygous de novo *CACNA1D* variants resulting in a loss of Cav1.3 activity are unlikely to cause human disease. This is strongly supported by previous findings both in knockout mice (for a review, see [3]) and Cav1.3-deficient humans with sinoatrial node dysfunction and deafness (SANDD; OMIM #614896 [24, 25]), in which functional loss of one or both *CACNA1D* alleles did not lead to a central nervous system (CNS) disease phenotype. This complicates the classification of new *CACNA1D* variants as high-risk mutations in genetic studies.

Here, we provide further convincing evidence for the high disease risk of gain-of-function de novo *CACNA1D* mutations for neurodevelopmental disorders. This is shown for mutation S652L, which has previously been identified in the Deciphering Developmental Disorders study, in a cohort of individuals with a severe developmental disorder of unknown cause [23]). However, in this study, it has not been classified as novel disease mutation with compelling evidence for pathogenicity and therefore *CACNA1D* has not been included as one of the 12 novel high-risk genes. Moreover, we demonstrate that a rare variant at the same position, S652W, induces a gating defect compatible with a loss-of-function, which explains its presence in apparently healthy individuals. Our data should raise awareness for the pathogenic potential of *CACNA1D* mutations, especially in patients without additional congenital endocrine symptoms as diagnostic features. De novo *CACNA1D* missense mutations may be underdiagnosed in clinical practice.

## Methods

### Complementary DNA constructs

Human wild-type (WT) Cav1.3 α_1_-subunits contained either exons 8a and 42 (WT_L_; long C-terminal splice variant; Genbank accession number: EU363339) or exons 8a and 43_S_ (WT_S_; short C-terminal splice variant [26];). All constructs were previously cloned into a pGFP^minus^ vector containing a CMV promoter, an ampicillin resistance gene and no GFP tag as described [26, 27].

Cloning of S652 constructs S652L_L_, S652L_S_, and S652W_L_: To introduce mutations S652L or S652W into various Cav1.3 splice variants SOE PCR was used. Briefly, nt 1685-4059 of WT_L_ or WT_S_ were PCR amplified with overlapping primers (primer pair 1 and 2) introducing the point mutations C>T (Ser>Leu) or C>G (Ser>Trp) at position nt 1967 in separate PCR reactions (PCR a and b) using WT_L_ or WT_S_ as templates. The two separate PCR products were then used as templates for the final PCR reaction (PCR c) with primer pair 3. This fragment was then AauI/HindIII digested and cloned into respective sites of WT_L_ or WT_S_ yielding hCav1.3_L_ S652L (S652L_L_) or hCav1.3_L_ S652W (S652W_L_) and hCav1.3_S_ S652L (S652L_S_). The following primer pairs were used for SOE PCR of S652L or S652W constructs (purchased from Eurofins MWG Operon, Ebersberg, Germany): primer pair 1, AauI (BrsGI) fwd: 5′-CCAACAAAGTCCTCTTGGCTCTGTTC-3′, S652L SOE rev: 5′-GATAATGAAGAGAAAAAGCAGAAGCAACAGC**A**AAGCGATGGACTTCATGGAGTTTAATAAG -3′ or S652W SOE rev: 5′-GATAATGAAGAGAAAAAGCAGAAGCAACAGC**C**AAGCGATGGACTTCATGGAGTTTAATAAG -3′ (314 bp); primer pair 2: S652L SOE fwd: 5′-CTTATTAAACTCCATGAAGTCCATCGCTT**T**GCTGTTGCTTCTGCTTTTTCTCTTCATTATC-3′ or S652W SOE fwd: 5′-CTTATTAAACTCCATGAAGTCCATCGCTT**G**GCTGTTGCTTCTGCTTTTTCTCTTCATTATC-3′, HindIII rev: 5′-ATAGATGAAGAACAGCATGGCTATGAGG-3′ (2122 bp); primer pair 3: AauI (BrsGI) fwd, HindIII rev (2375 bp).

PCR reaction mix for PCR a, b and c contained 5 μl 10x Pfu buffer with 20 mM MgSO_4_ (Cat# EP0571; Thermo Fisher Scientific,Waltham, MA, USA), 2 mM dNTP mix (Cat# R0241; Thermo Fisher Scientific, Waltham, MA, USA), 2.5 μl DMSO, 500 ng DNA template (PCR a and b) or 0.5 μl of PCR products a and b (PCR c), 10 pMol/μl forward primer, 10 pMol/μl reverse primer, 0.5 μl Pfu polymerase (native) (2.5 units/μl; Cat# EP0571; Thermo Fisher Scientific, Waltham, MA, USA) and nuclease-free water to a final volume of 50 μl. Following PCR program was performed: initial denaturation at 95 °C for 3 min, then 35 cycles of 30 s denaturation at 95 °C, 30 s annealing at 50 °C, variable extension time at 72 °C depending on fragment size (for Pfu polymerase: 2 min/kb) followed by a final elongation step of 72 °C for 7 min. The integrity of all cloned constructs was confirmed by restriction site mapping and sequencing (Eurofins MWG Operon, Ebersberg, Germany).

### Cell culture and transfection

For whole-cell patch-clamp recordings, tsA-201 cells (a human embryonic kidney (HEK)-293 subclone stably expressing SV40 temperature-sensitive T-antigen, ECACC, 96121229) were cultured in Dulbecco’s modified Eagle’s medium (DMEM; Cat# D6546; Merck KGaA, Darmstadt, Germany) containing 4500 mg/l L-glucose, 10% fetal bovine serum (FBS; Cat# 10270106; Thermo Fisher Scientific, Waltham, MA, USA), 2 mM L-glutamine (Cat# 25030032; Thermo Fisher Scientific, Waltham, MA, USA), 10 units/ml penicillin G (Cat# P-3032; Merck KGaA, Darmstadt, Germany), 10 μg/ml streptomycin (Cat# S-6501; Merck KGaA, Darmstadt, Germany) and maintained at 37 °C in a humidified incubator with 5% CO_2_. Cells were grown to ~ 80% confluency and split using 0.05% trypsin for cell dissociation. Cells were transiently transfected using the Ca^2+^-phosphate precipitation method always including EGFP (1.5 μg) as a transfection marker. For recordings of WT_L_ vs S652L_L_ or S652W_L_ tsA-201 cells were transiently transfected with human ɑ_1_ (3 μg), rat β_3_ (2 μg; Genbank accession number NM_012828), and rabbit ɑ_2_δ-1 (2.5 μg, Genbank accession number NM_001082276) subunits whereas for recordings of WT_S_ vs S652L_S_ HEK-293 cells stably expressing β_3_ and ɑ_2_δ-1 were used and required only LTCC ɑ_1_ (3 μg) transient transfection [26, 28, 29]. HEK-293 cells stably expressing β_3_ and ɑ_2_δ-1 were periodically treated with selection agents for each subunit (β_3_, 500 μg/ml geneticin (Cat# 10131027; Thermo Fisher Scientific, Waltham, MA, USA); ɑ_2_δ-1, 10 μg/ml blasticidin S HCl (Cat# A1113903; Thermo Fisher Scientific, Waltham, MA, USA)). All data were obtained from > 3 independent transfections. On the following day, cells were trypsinized (0.05% trypsin) and plated onto poly-l-lysine-(Cat# P-2636; Merck KGaA, Darmstadt, Germany) precoated 35-mm culture dishes. Cells were kept at 30 °C and 5% CO_2_ and were subjected to electrophysiological experiments 20–72 h after transfection.

### Electrophysiological recordings in tsA-201 cells

**Fig. 1:**
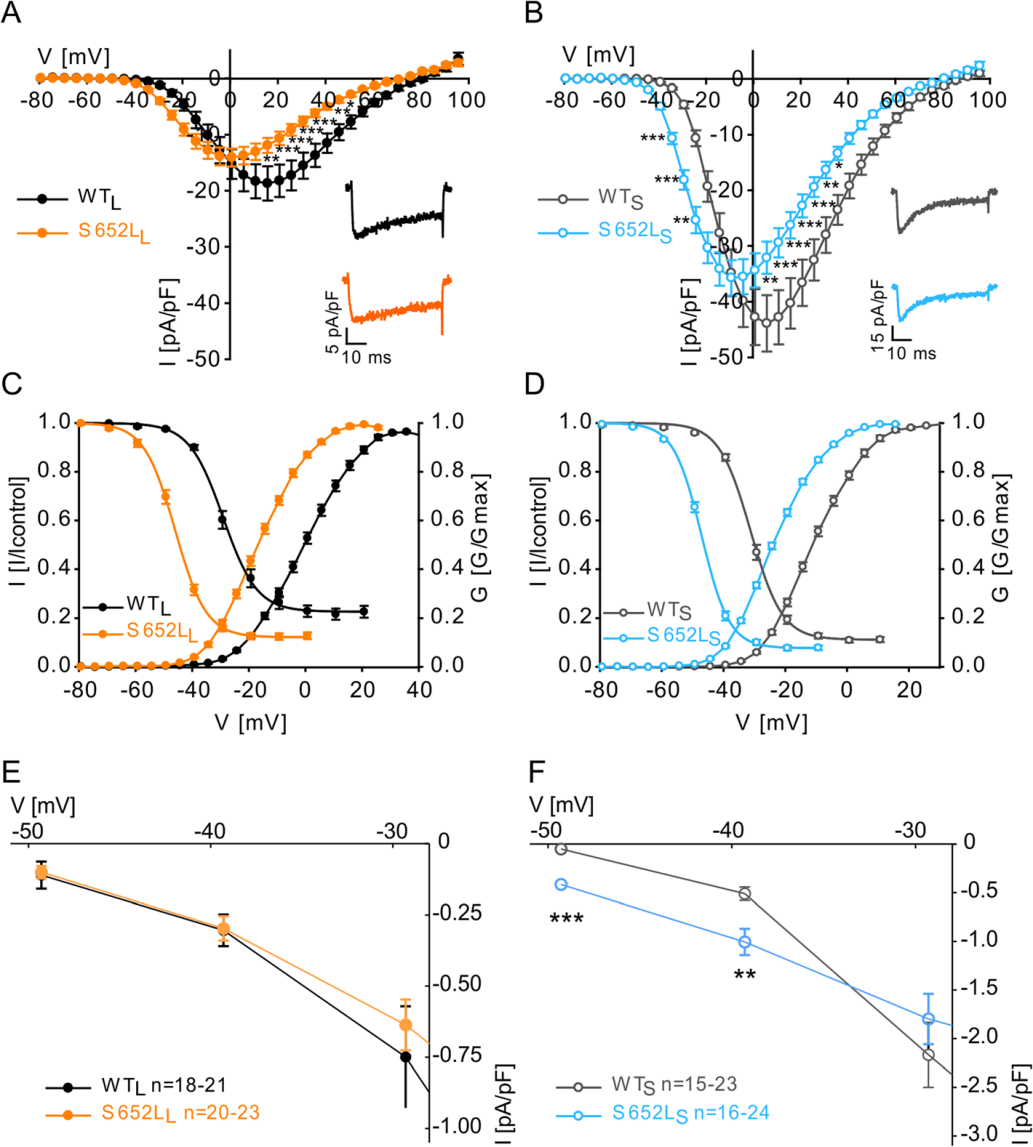
Mutation S652L induces severe gating changes. **a**, **b** Current-voltage relationship (*I*_Ca_; mean ± SEM) of WT and mutant C-terminal long (WT_L_, S652L_L_, A) and short (WT_S_, S652L_S_, B) Cav1.3 splice variants recorded in parallel on the same day using 50-ms depolarizing square pulses to various test potentials from a holding potential (HP) of -89 mV. Inset: Representative *I*_Ca_ traces upon depolarization to the potential of maximal inward current (*V*_max_). Statistics: two-way ANOVA followed by Bonferroni post hoc test, *p < 0.05, **p < 0.01, ***p < 0.001. **c**, **d** Normalized steady-state activation and inactivation curves of WT_L_ vs S652L_L_ (**c**) and WT_S_ vs S652L_S_ (**d**). Data are presented as mean ± SEM; for parameters, statistics and number of experiments see Table 1. **e, f** Window currents of WT_L_ vs S652L_L_ (E) and WT_S_ vs S652L_S_ (F). Data were obtained by multiplying the steady-state inactivation (**c, d**) at a given potential with the corresponding current densities of the I-V-relationships (**a, b**). Statistics: Student's t-test (multiple comparison adjusted),***p < 0.001, **p < 0.01. Data are represented as mean ± SEM for the *n*-numbers indicated. Data were collected from > 3 independent transfections

For whole-cell patch-clamp experiments, patch pipettes were pulled in a micropipette puller (Sutter Instrument, Novato, CA, USA) using borosilicate glass capillaries (borosilicate glass; Cat# 64-0792, Warner Instruments, Hamden, CT, USA) and fire-polished using a MF-830 microforge (Narishige Co, Tokyo, Japan). Pipettes with a resistance of 1.5–3 MΩ were backfilled with internal solution containing (in mM): 135 CsCl, 10 Cs-EGTA, 1 MgCl_2_, 10 HEPES, 4 Na_2_ATP adjusted to pH 7.4 with CsOH. The bath solution contained (in mM): 15 CaCl_2_ or 15 BaCl_2_, 150 Choline-Cl, 1 MgCl_2_, 10 HEPES, adjusted to pH 7.3 with CsOH. Whole-cell patch-clamp recordings were performed at room temperature (20-23 °C) using an Axopatch 200B Amplifier (Molecular Devices, San José, CA, USA). Data were digitized (Digidata, 1322A digitizer, Molecular Devices, San José, CA, USA) at 50 kHz, low-pass filtered at 1–5 kHz and analyzed using pClamp 10.2 software (Molecular Devices, San José, CA, USA). Series resistance was compensated by 60–90% and all voltages were corrected for a liquid junction potential of − 9.3 mV [28]. Currents were leak subtracted either offline using a 50-ms hyperpolarizing voltage step from − 89 to − 99 mV or using an online P/4 protocol. Current-voltage (*I*-*V*) relationships were measured by applying 50 ms depolarizing square pulses to various test potentials (Δ 5 mV increments) starting from a holding potential (HP) of − 89 mV. *I*-*V* curves were fitted to the equation *I* = G_max_ (*V* − *V*_rev_)/(1 + exp [− (*V* − *V*_0.5_)/k]) where *I* is the peak current, *G*_max_ is the maximum conductance, *V* is the test potential, *V*_rev_ is the extrapolated reversal potential, *V*_0.5_ is the half-maximal activation voltage, and k is the slope factor. The voltage dependence of activation was obtained from the *I*-*V* relationship by calculating the conductance (*G* = *I*/*V* − *V*_rev_) followed by normalization (*G*/*G*_max_) and plotting as a function of voltage. The G-V curve was fitted using the following Boltzmann relationship: *G* = *G*_max_/(1 + exp[− (*V* – *V*_0.5_)/*k*]. The steady-state inactivation was determined by calculating the ratio between current amplitudes of a control versus a test pulse (I/I_control_; both 20 ms to *V*_max_) separated by a 5-s conditioning step to various potentials (10 mV increments; 30 s intersweep interval; HP: − 89 mV) and plotting as a function of voltage. Steady-state inactivation curves were fitted using a modified Boltzmann equation: *G* = (1 – *G*_max_)/(1 + exp [(*V* – *V*_0.5,inact_)*/k*_inact_] + *G*_max_ where *V*_0.5inact_ is the half-maximal inactivation voltage and *k*_inact_ is the inactivation slope factor. Channel open probability was estimated by dividing the peak ionic tail current (*I*_tail_) by the integrated “ON” gating charge (Q_ON_) at the potential where there is no ionic inward or outward current (*V*_rev_). ON-gating currents were filtered at 5 kHz and digitized at 50 kHz. Window currents were obtained by multiplying the steady-state inactivation at a given voltage (Fig. 1c,d, Fig. 6b) with the corresponding current densities (pA/pF) at the given potentials of the *I*-*V* relationships (Fig. 1a,b, Fig. 6a). Pulses to physiologically relevant potentials i.e. − 20 mV which corresponds to ∼ − 35–38 mV in physiological Ca^2+^-concentrations were applied for 5-s. Normalized inward Ca^2+^-currents (*I*_Ca_) were multiplied with the corresponding conductance at − 20 mV (*G*: WT_L_, 0.1253; S652L_S_, 0.4330; WT_S_, 0.2647; S652L_S_, 0.6325) and plotted as a function of time. The percentage of inactivation during a 5-s long depolarizing pulse from a HP of − 89 mV to the potential of maximal inward current (*V*_max_) was determined after 50, 100, 250, 500, 1000, and 5000 ms with Ca^2+^ or Ba^2+^ as a charge carrier. Ca^2+^-dependent inactivation (CDI) was determined over a broad voltage range by analyzing the fraction of remaining currents at the end of 250 ms depolarizations (expressed as fraction of the peak current amplitude, *r*_250_) to different test potentials (∆ 10-mV increments) with Ca^2+^ or Ba^2+^ as a charge carrier resulting in typical U-shaped dependence of voltage. The difference between Ca^2+^- and Ba^2+^-curves indicates the strength of CDI and is defined as parameter f for each voltage step and plotted as a function of voltage. The fractional Ca^2+^-dependent component of inactivation after 250 ms was calculated as CDI = 1 − *r*_Ca_/*r*_Ba_, where *r*_Ca_ and *r*_Ba_ is the fraction of current remaining at a given time point of inactivation, respectively. Persistent currents were determined after 5-s long depolarizations to different potentials expressed as fractional persistent current (%) normalized to the peak current amplitude measured by a 20-ms pre-pulse to the *V*_max_ in the same sweep. Tail currents were obtained from a transition from + 80 mV to − 60 mV or − 40 mV and normalized tail currents were fitted to a bi-exponential equation. In general, experiments with currents < 100 pA and > 1000 pA (range was defined prospectively) were excluded from analysis to avoid errors in the measurements of *V*_0.5,act_, which tends to become more hyperpolarized at larger current amplitudes. For pharmacological experiments, cells were depolarized using a 100-ms square pulse to the *V*_max_ of each individual cell (0.1 Hz; HP: − 89 mV). Cells were perfused using an air pressure-driven perfusion system (BPS-8 Valve Control System, ALA Scientific Instruments) with external bath solution (15 mM Ca^2+^) in the presence or absence of isradipine (Fisher scientific, 50-850-70001) with a flow rate of 0.5 ml/min. Isradipine stocks were prepared in DMSO and freshly diluted 1:1000 in bath solution to the final concentration prior to the experiment. On each recording day, individual control recordings with bath solution only were performed using the same tubes subsequently used for isradipine experiments. Drug application was started after at least three constant control sweeps during perfusion with bath solution. Drug effects were corrected for linear current decay (“run-down”) measured in control cells.

### Ca^2+^-imaging

HEK-293 cells stably expressing β_3_ and α_2_δ-1 were transfected with WT_L_ or S652L_L_ Cav1.3 α_1_-subunits (3 μg) together with EGFP (1.5 μg) for visualization of transfected cells. Cells were patched 24–72 h after transfection at room temperature (21–23 °C) with internal solution containing (in mM): 114 CsMeSO_3_, 5 CsCl, 1 MgCl_2_, 4 Na_2_ATP, 10 HEPES and 0.5 Cs-EGTA (pH 7.3) and 0.2 mM Fluo-4 pentapotassium salt (cat#: F14200, Thermo Fisher). During the recording, cells were kept in Tyrode’s solution containing (in mM): 135 NaCl, 5.4 KCl, 1.8 CaCl_2_, 0.33 MgCl_2_, 0.33 NaH_2_PO_4_, 5 HEPES and 5 glucose (pH 7.4). Fluorescence from transfected cells was detected using Photon Technology International (PTI) photomultipliers and manufacturer’s software. Excitation was performed at 488 nm and fluorescent emission from each sample was recorded at 520 nm. Action potential waveform (APW)-like stimulus trains were applied at a frequency of 10 Hz and 300 sweeps per run. The APW protocol was elicited from – 80-mV HP composed of 3 voltage ramps: step: − 80 to − 60 mV for 2.5 ms, 1st ramp: − 60 to + 20 mV in 1 ms, 2nd ramp: + 20 to - 70 mV in 1.5 ms, 3rd ramp: − 70 to − 60 mV in 5 ms (afterhyperpolarization), step: − 60 mV for 90 ms. Fluorescent signals were normalized to baseline fluorescence (F_0_) and current density (pA/pF) determined in a ramp before the start of the train. Ca^2+^-charge of WT_L_ and S652L_L_ was obtained by integrating the area of *I*_tail_ normalized to maximum *I*_Ca_ determined in a ramp before the start of the train.

### Protein preparation and immunoblot analysis of HEK-293 cells

HEK-293 cells stably expressing β_3_ and ɑ_2_δ-1 were transiently transfected with WT_L/S_ and mutant α_1_-subunits using JetPrime® transfection reagent (VWR International, Radnor, PA, USA) according to the manufacturer’s protocol. Membrane preparations were performed 48 h after transfection by first washing cells with phosphate-buffered saline (in mM: 137 NaCl, 2.7 KCl, 8 Na_2_HPO_4_, 1.5 KH_2_PO_4_). Subsequently, cells were harvested and resuspended in 2 ml lysis buffer (10 mM Tris-HCl, 1 μg/ml aprotinin, 0.1 mg/ml trypsin inhibitor, 1 μM pepstatin A, 0.5 mM benzamidine, 0.2 mM phenylmethylsulfonylfluoride, 2 mM iodacetamide, 1 μl/ml leupeptin, pH 7.4) and lysed on ice for 15 min. After resuspension, the mixture was homogenized by passing it through a 27-gauge cannula and centrifuged for 20 min at 726×*g* to remove cell debris. Membranes were collected by subjecting the resulting supernatant to an ultracentrifugation step at 110,561×*g* for 30 min. The pellet was resuspended in 200 μl of lysis buffer and stored at − 80 °C. Protein concentrations were measured via Bradford assay. Proteins were mixed with 4× NuPAGE^TM^ LDS sample buffer (Cat# NP0008; Thermo Fisher Scientific, Waltham, MA, USA) and incubated at 70 °C for 10 min before loading onto NuPAGE^TM^ 3–8% Tris-acetate protein gels (Thermo Fisher Scientific, Waltham, MA, USA) together with a PageRuler^TM^ Plus prestained protein ladder (10-250 kDa; Cat# 26619; Thermo Fisher Scientific, Waltham, MA, USA). Proteins were separated using a 20× NuPAGE^TM^ Tris-acetate SDS running buffer (Cat# LA0041; Thermo Fisher Scientific, Waltham, MA, USA) at 50 V for 40 min and 100 V for 1 h. Proteins were transferred onto a polyvinyliden fluoride membrane (PVDF, Immobilon-P membrane, Cat# IPVH00010; Merck KGaA, Darmstadt, Germany) in transfer buffer (20x NuPAGE^TM^ Tris-acetate SDS running buffer, 20% Methanol (v/v)) at 100 V for 90 min. After the transfer, the membrane was blocked for 2 h at room temperature in blocking buffer (5% milk powder and 0.2% gelatine in 20 mM Tris, 150 mM NaCl, 0.5% Triton X-100, 0.1% Tween 20) and incubated with the primary antibody in blocking buffer at 4 °C overnight. α_1_-subunits were detected using anti-Cav1.3 antibody (rabbit-polyclonal; Cat# ACC-005, Lot# AN2150; Alomone labs, Jerusalem, Israel; diluted 1:100,000). Anti-ɑ-tubulin (mouse-monoclonal (DM1A); Cat# CP06, Lot# D00143511; Merck KGaA, Darmstadt, Germany; diluted 1:100,000) was used as loading control. After washing with washing buffer (20 mM Tris, 150 mM NaCl, 0.5% Triton X-100, 0.1% Tween 20) the membrane was incubated with the secondary antibody in blocking buffer for 2 h at room temperature followed by another wash step. Peroxidase conjugated goat anti-rabbit IgG (whole molecule; Cat# A0545, Lot# SLBC3108; Merck KGaA, Darmstadt, Germany; diluted 1:15,000) and peroxidase conjugated goat anti-mouse IgG (H+L; Cat# 31430, Lot# SC245915; Thermo Fisher Scientific, Waltham, MA, USA; diluted 1:5000) were used as secondary antibodies. For detection, the SuperSignal^TM^ West Femto maximum sensitivity substrate (Cat# 34096; Thermo Fisher Scientific, Waltham, MA, USA) was used. Signals were quantified with a FUSION FX7 Imager (Vilber Lourmat Deutschland GmbH, Eberhardzell, Germany) and analysis of band intensity using ImageJ 1.46 (National Institute of Health). Integrated densities of mutant and WT signals were normalized to the loading control.

The absolute expression level of α_1_-subunit protein varies between membrane preparations of individual transfections. Therefore, transfections, membrane preparations, and Western blot analysis were always carried out in parallel for WT and mutant channels.

### Homology modeling

We predicted the structure of the WT Cav1.3 α_1_-subunit and two mutants by developing a homology model based on the cryo-electron microscopy (EM) structure of the Cav1.1 α_1_-subunit in the closed (potentially inactivated) state [30]. The high sequence conservation of the Cav1.1 α_1_-subunit to the Cav1.3 α_1_-subunit (~ 75% similarity and ~ 62% identity measured by MOE) allowed us to predict a reliable structure model.

Homology modeling has been performed using Rosetta and MOE (Molecular Operating Environment, version 2018.08, Molecular Computing Group Inc., Montreal, Canada). Additionally, ab initio Rosetta was used to generate structures for loops that were not resolved in the original Cav1.1 α_1_-subunit template. The structures for the mutants were derived from the WT model by replacing the mutated residue and carrying out a local energy minimization using MOE. The C-terminal and N-terminal parts of each domain were capped with acetylamide (ACE) and N-methylamide to avoid perturbations by free charged functional groups. The structure model was embedded in a plasma membrane consisting of POPC (1-palmitoyl-2-oleoyl-sn-glycero-3-phosphocholine) and cholesterol in a 3:1 ratio, using the CHARMM-GUI Membrane Builder. Water molecules and 0.15 M KCl were included in the simulation box. Energy minimizations of WT and mutant structures in the membrane environment were performed. The topology was generated with the LEaP tool of the AmberTools18, using force fields for proteins and lipids, ff14SBonlysc and Lipid14, respectively. The WT and mutant structures were gradually heated from 0 to 300 K in two steps, keeping the lipids fixed, and then equilibrated over 1 ns. Then molecular dynamics simulations were performed for 10 ns, with time steps of 2 fs, at 300 K and in anisotropic pressure scaling conditions. Van der Waals and short-range electrostatic interactions were cut off at 10 Å, whereas long-range electrostatics were calculated by the Particle Mesh Ewald (PME) method. MOE was used to visualize the key interactions and point out differences in the WT and mutant structures.

### Ethics approval

The Deciphering Developmental Disorders Study [23] has UK Research Ethics Committee approval (10/H0305/83, granted by the Cambridge South REC, and GEN/284/12 granted by the Republic of Ireland REC). Parental informed consent has been obtained for updated clinical information of the probands.

### Statistics

Data were analyzed using Clampfit 10.2 (Axon Instruments) and Sigma Plot 11 (Systat Software, Chicago, IL). For statistical analysis Graph Pad Prism 5.01 software (GraphPad Software, La Jolla, CA) was used. Significance of group differences between two groups was determined using unpaired Student’s *t* test for normally distributed data or Mann-Whitney *U* test for non-normally distributed data. Significance of group differences between three and more groups was determined using one-way analyses of variance (ANOVA) or two-way ANOVA for normally distributed data (with Bonferroni post-test as indicated). All data are represented as mean ± SEM. Significance level was set to α-error lower than *p* < 0.05 (*), *p* < 0.01 (**) and *p* < 0.001 (***). All original datasets are available from the corresponding author on reasonable request.

## Results

### S652 L, a novel *CACNA1D* de novo mutation in monozygotic twins with a severe neurodevelopmental disorder and ASD

The novel *CACNA1D* variant has been reported in the Deciphering Developmental Disorders Study [23]. In a large unbiased genotype-driven approach this study analyzed 1,133 children with severe, undiagnosed developmental disorders and their parents using a combination of exome sequencing and array-based detection of chromosomal rearrangements [23]. The *CACNA1D* variant (chr3: 53757881 C>T, human reference genome hg19) was predicted to cause a p.Ser652Leu (S652L) mutation (reference sequence NM_001128839). However, it was not considered a novel disease mutation in this study. The patients (DECIPHER database individuals #262954 and #262956; decipher.sanger.ac.uk) are male monozygotic twins, thirteen years old at present, and both harbor one copy of the mutation. Their clinical phenotype has been updated recently. It manifests as a severe neurodevelopmental phenotype with delayed speech and language development and a global developmental delay. Both individuals show self-injurious behaviors and have been diagnosed with ASD two years ago. One patient is tall (5 ft 5 in.) and heavy (57 kg) for his age, has undescended testes and suffered from seizures at the age of two without recurrence. In addition, he shows challenging behavior with attention deficit hyperactivity disorder-like symptoms. No abnormal blood pressure has so far been reported and both are currently not treated with any medication. In addition, facial dysmorphism characterized by epicanthus, abnormality of the nose, microtia, a small vermillion border, and widely spaced teeth was noted. Prediction of the possible impact of this amino acid substitution on protein structure and function using bioinformatics prediction tools PolyPhen2, SIFT and MutationTaster indicated a probably damaging (score: 1.00), deleterious (100%) or disease-causing (probability: 0.999) effect, respectively. This variant is not reported in the gnomAD database (a reference database that lists exomes and genomes from a total of 141,456 unrelated individuals harboring mutations without pediatric disease [21]).

A frameshift mutation (c.1934_1935insG (p.Glu646GlyfsTer)) in the *KIF22* gene (chr16: 29816479 T>TG, human reference genome hg19) was also identified in both patients. This gene is highly expressed in bones, cartilage, skin, ligaments and joint capsules [31]. Mutations in the *KIF22* gene have so far not been associated with neurodevelopmental disorders but result in a syndrome called spondyloepimetaphyseal dysplasia with joint laxity (SEMDJL; OMIM #603213), with malformations of the spine, skeletal dysplasia and malalignment of limbs but no intellectual impairment. Complete knock-out of *Kif22* in mice results in premature intrauterine death but surviving *Kif22*^−/−^ embryos develop into healthy adult mice [32]. Moreover, protein loss-of-function variants (stop gained, frameshift mutations) are reported in more than 40 controls in the gnomAD database. Therefore, the *KIF22* mutation is unlikely to explain the severe neurodevelopmental phenotype of the two patients. In contrast, *CACNA1D* mutation S652L has also been reported as a somatic aldosterone-producing-adenoma (APA) mutation [33] in a patient suffering from resistant hypertension, providing additional indirect evidence for a pathogenic role of this novel germline *CACNA1D* variant.

### Mutation S652L changes the voltage-dependence of activation and inactivation

Based on recent findings of unique gating changes induced by pathogenic *CACNA1D* de novo mutations [14–18, 20, 34], we therefore hypothesized that S652 L could also explain the neurodevelopmental phenotype in both patients. For a detailed biophysical characterization in tsA-201 cells we introduced this mutation into the biophysically distinct C-terminally long (WT_L_, S652L_L_) and short (WT_S_, S652L_S_) Cav1.3 splice variants [26]. Both splice variants are abundantly expressed in the brain and differ considerably with respect to their biophysical properties, with higher voltage-sensitivity and faster Ca^2+^-dependent inactivation of WT_S_ [3, 16]. Mutant α_1_-subunit proteins were expressed as intact proteins with the expected molecular mass (Additional file 1: Figure S1).

Mutation S652L induced pronounced gating changes. It significantly shifted the voltage-dependence of activation (Fig. 1a, b) and steady-state inactivation to more negative potentials indicating a phenotype that can support a channel gain-of-function in both splice variants (Fig. 1c, d; for parameters see Table 1) by promoting Ca^2+^-inward currents (I_Ca_) at negative voltages. As a consequence the mutation induced a higher window current at subthreshold potentials (− 50 and − 40 mV) compared to WT in the short Cav1.3 splice variant (Fig. 1e, f). These variants comprise about half of the Cav1.3 α_1_-subunits in the brain [35]. At − 50 mV significant window current was only measurable in S652 L_S_ but not in WT_S_, and it was two times larger in the mutant at − 40 mV. Whereas current amplitudes were larger at negative voltages, *I*_Ca_ above the potential of maximal inward current (*V*_max_) were significantly smaller (Fig. 1a, b). Since gating currents, which are a measure for the number of active channels at the cell surface, were not different in the mutant channels (Q_ON_ [mean ± SEM; pA*ms)]: WT_L_, 158.9 ± 26.3, *n* = 23; S652 L_L_, 140.3 ± 25.1, *n* = 21; Mann-Whitney test), reduced current density is likely due to a decreased open probability (P_O_). This is further supported by a significantly decreased slope of the *I*_tail_/Q_ON_ relationship for S652 L_L_ channels (*I*_tail_/Q_ON_ [linear regression slopes, mean ± SEM; ms^−1^]: WT_L_: − 7.22 ± 0.916, *r*^2^ = 0.72, *n* = 26; S652 L_L_: − 4.24 ± 0.657, *r*^2^ = 0.72, *n* = 25; slopes are significantly different: *F* = 6.43, *p* = 0.015, *F* test; Additional file 2: Figure S2).

**Table 1:** Steady-state activation and inactivation parameters of mutation S652L

	Activation				Inactivation			
α_1_-subunit	*V*_0.5_ (mV)	*k* (mV)	*V*_rev_ (mV)	*n*	*V*_0.5,inact_ (mV)	*k* (mV)	Non-inactivating (%)	*n*
WT_L_	− 0.18 ± 0.97	9.63 ± 0.19	71.1 ± 1.46	21	− 25.7 ± 1.42	5.64 ± 0.26	22.5 ± 2.26	18
S652L_L_	− 16.3 ± 0.75***	8.27 ± 0.10***	59.9 ± 1.03***	23	− 43.3 ± 1.29***	4.99 ± 0.12*	12.8 ± 1.31***	20
WT_S_	− 10.6 ± 0.72	7.65 ± 0.13	64.7 ± 0.71	23	− 31.2 ± 0.67	4.78 ± 0.23	12.1 ± 0.99	15
S652L_S_	− 23.5 ± 0.44***	7.32 ± 0.10*	58.4 ± 0.54***	24	− 47.2 ± 0.42***	4.24 ± 0.16	9.14 ± 0.96*	15

Parameters were obtained from fitting normalized steady-state activation (*G*/*G*max) or inactivation curves (*I*/*I*max) to a Boltzman relationship. All values are presented as mean ± SEM (> 3 independent transfections). Statistics: unpaired Student’s *t* test, *p < 0.05, ***p<0.001 compared to WT. *n*, number of recordings. *V*_0.5_, half maximal activation/inactivation voltage; *V*_rev_, reversal potential; *WT*, wild-type

Note that short Cav1.3 splice variants have very small, non-measurable ON-gating currents [26] and were therefore not further analyzed here.

### Mutation S652L accelerates voltage-dependent inactivation but has opposing effects on Ca^2+^-dependent inactivation

**Fig. 2:**
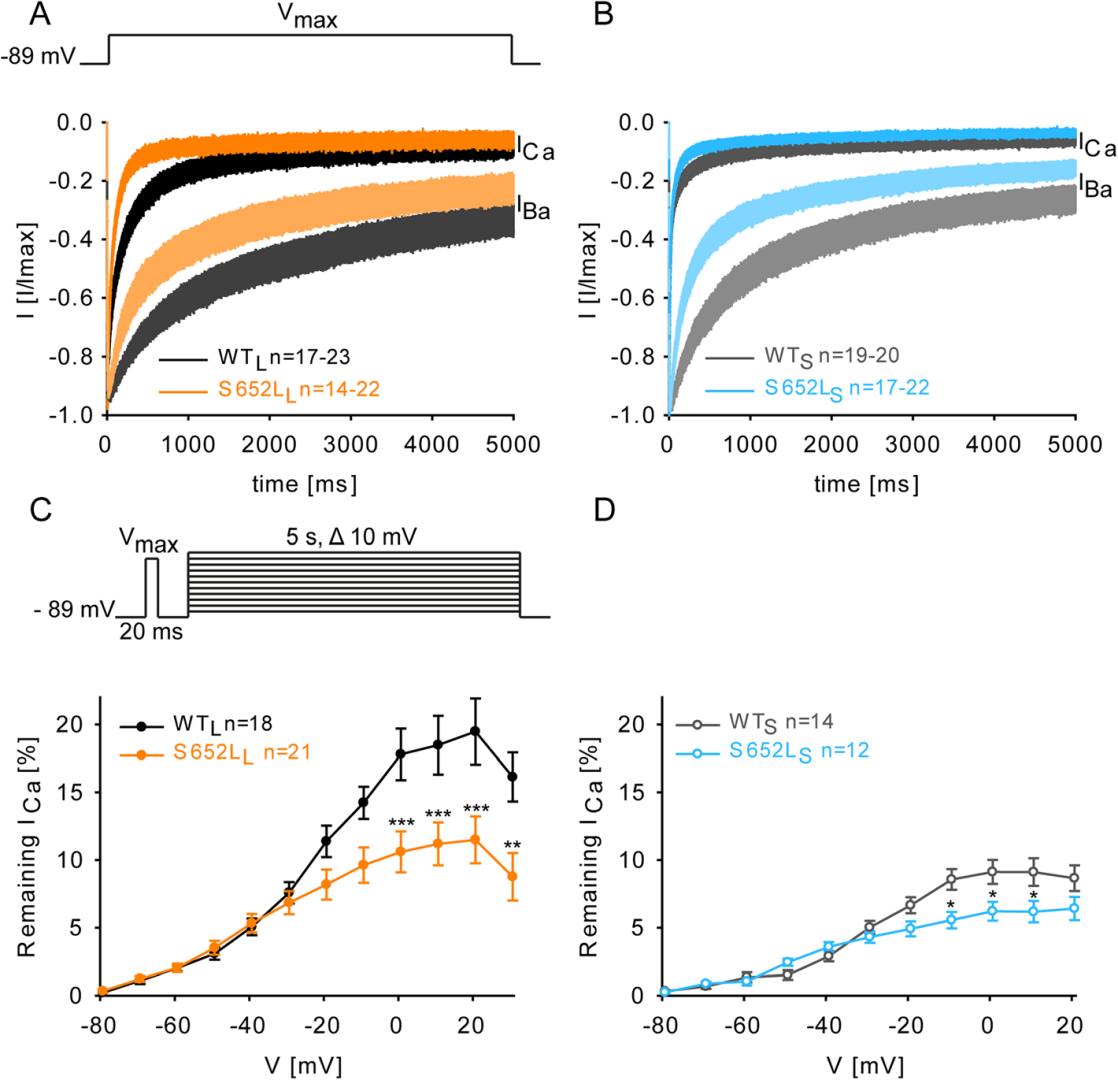
Mutation S652L accelerates voltage-dependent inactivation and reduces persistent currents. (**a**, **b**) Inactivation kinetics of WT_L_ vs S652L_L_ (A) and WT_S_ vs S652L_S_ (B) during a prolonged (5-s) depolarization to *V*_max_ with Ca^2+^ (CDI and VDI) or Ba^2+^ (VDI only; dim colors) as a charge carrier. Data are presented as mean ± SEM for the *n*-numbers indicated; for statistics see Table 2. **c, d** Persistent currents were determined after 5-s long depolarizations to different potentials and are expressed as % of the peak current amplitude measured by a preceding 20 ms pre-pulse to *V*_max_. Statistics: two-way ANOVA followed by Bonferroni post hoc test, *p < 0.05, ***p < 0.001. Data are represented as mean ± SEM for the *n*-numbers indicated.

**Fig. 3:**
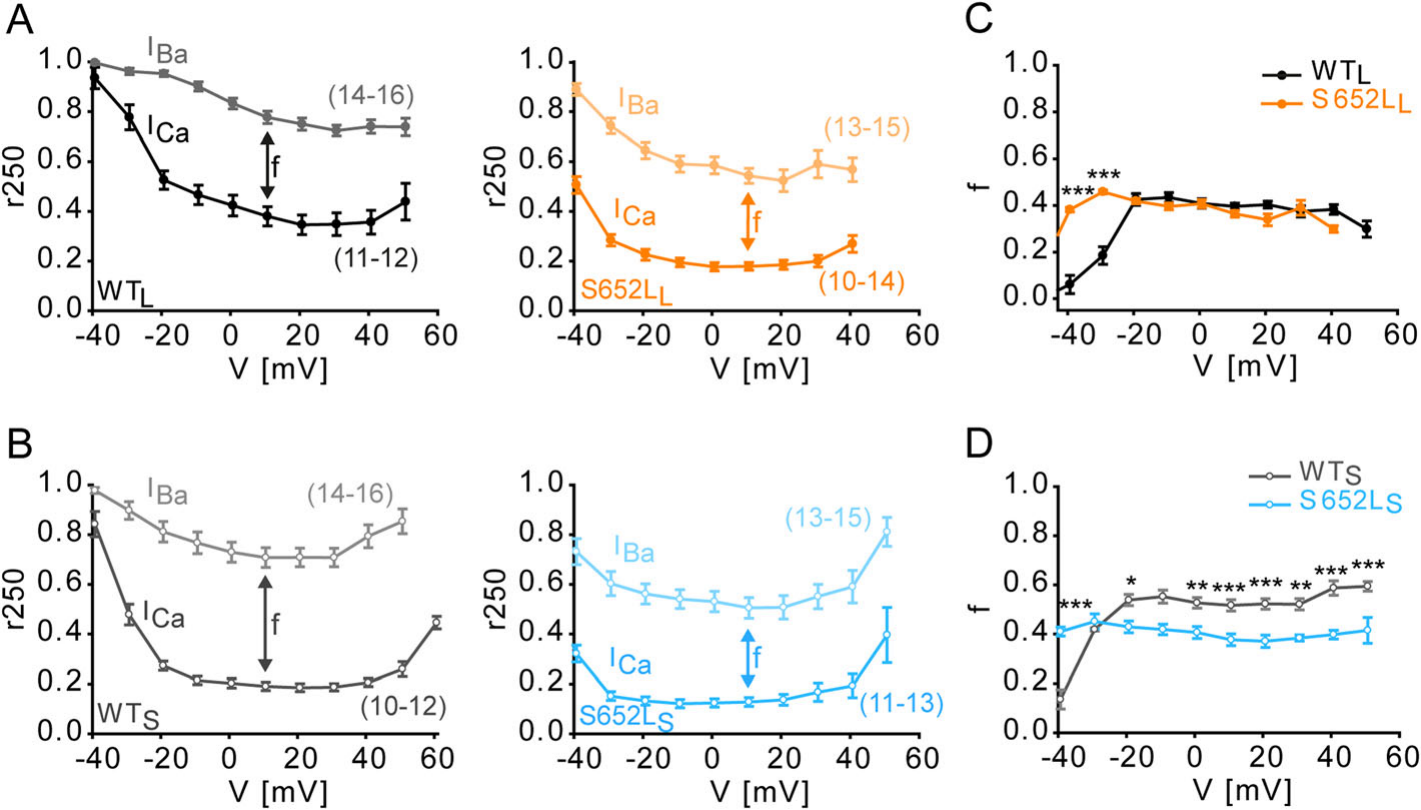
Mutation S652 L alters Ca^2+^-dependent inactivation over a broad voltage range. **a, b** Voltage-dependence of Ca^2+^-dependent inactivation of WT_L_ vs S652L_L_ (A) and WT_S_ vs S652L_S_ (B). The fraction of remaining currents was determined at the end of 250 ms (r_250_) upon depolarizations to different test potentials resulting in a typical U-shaped dependence of voltage. Data are presented as mean ± SEM (> 3 independent transfections); *n*-numbers are given in parenthesis. **c, d** *f* values over a broad voltage range for WT_L_ vs S652L_L_ (C) and WT_S_ vs S652L_S_ (D). *f* is defined as the difference between r_250_ values of *I*_Ba_ and *I*_Ca_ at each voltage step and indicates the strength of CDI. CDI of S652 L_L_ and S652L_S_ was more pronounced at negative potentials. At higher potentials CDI remained unchanged for the long mutant but was significantly reduced for S652L_S_. Statistics: two-way ANOVA of *f* values followed by Bonferroni post hoc test, *p < 0.05, **p < 0.01, ***p < 0.001 compared to WT. Data are presented as mean ± SEM (> 3 independent transfections)

Since either acceleration or slowing of the inactivation time course of Cav1.3 is also a hallmark of pathogenic *CACNA1D* mutations, we studied voltage- (VDI) and Ca^2+^-dependent inactivation (CDI). Mutation S652L significantly accelerated inactivation kinetics (Fig. 2) during 5-s depolarizations to *V*_max_ with both Ba^2+^ (which reports VDI) and Ca^2+^ (which, in addition, induces CDI) as charge carriers in both C-terminally long (Fig. 2a) and short (Fig. 2b; for statistics, see Table 2) splice variants. It also significantly reduced *I*_Ca_ during long-lasting depolarizations as shown as the percentage of remaining current after 5-s depolarizations to various test potentials in both Cav1.3 splice variants (Fig. 2c, d). By comparing the difference of fractional inactivation between I_Ca_ and inward Ba^2+^-current (*I*_Ba_) after 250-ms depolarizations to different test potentials (*f* value, see Fig. 3) mutational effects on the voltage-dependence of CDI could also be determined. Whereas maximal CDI was unchanged in the long Cav1.3 splice variant (Fig. 3a, c), it was significantly reduced in the short variant (Fig. 3b, d). Therefore, faster inactivation of I_Ca_ must be due to the acceleration of VDI, despite being partially compensated by reduced CDI in WT_S_. Assuming that CDI and VDI are independent processes, we also calculated the fractional Ca^2+^-dependent component of inactivation as previously described [36] for WT_S_ vs S652L_S_ from the data shown in Table 2. After 250 ms of inactivation there was no difference between WT_S_ (0.778 ± 0.027) and S652L_S_ (0.805 ± 0.021; *p* = 0.40, *n* = 21; unpaired Student’s *t* test) and this was also true for all other time points. This further confirms that S652L can promote Cav1.3 inactivation largely by affecting VDI.

**Table 2:** Normalized inactivation kinetic parameters of mutation S652L

α_1_-subunit	*r*_50_	*r*_100_	*r*_250_	*r*_500_	*r*_1000_	*r*_5000_	*n*
Remaining I_Ca_ [%]							
WT_L_	65.6 ± 2.66	54.5 ± 2.96	36.9 ± 2.96	24.9 ± 2.65	16.9 ± 2.40	8.60 ± 2.20	23
S652L_L_	52.8 ± 2.05***	35.2 ± 1.92***	17.1 ± 1.68***	10.8 ± 1.52***	8.04 ± 1.43**	5.33 ± 1.33	22
WT_S_	29.3 ± 2.07	23.3 ± 1.90	17.1 ± 1.71	12.9 ± 1.45	10.1 ± 1.10	6.21 ± 0.81	20
S652L_S_	26.4 ± 1.47	17.6 ± 1.25*	10.5 ± 0.99**	7.11 ± 1.05**	6.15 ± 0.63**	3.34 ± 0.40**	22
Remaining I_Ba_ [%]							
WT_L_	92.55 ± 0.77	88.74 ± 1.02	79.10 ± 1.77	68.4 ± 2.75	57.37 ± 3.36	30.26 ± 3.30	17
S652L_L_	86.78 ± 0.92***	76.91 ± 1.70***	59.05 ± 2.42***	47.17 ± 2.94***	36.33 ± 3.12***	17.24 ± 2.70**	14
WT_S_	93.57 ± 0.88	88.24 ± 1.46	76.96 ± 2.83	64.48 ± 3.64	50.89 ± 4.26	25.67 ± 3.49	19
S652L_S_	84.74 ± 1.66***	72.67 ± 2.70***	53.91 ± 3.35***	40.74 ± 3.35***	29.98 ± 2.76***	15.27 ± 1.92*	17

*r* values represent the percent of remaining *I*_Ca_ or *I*_Ba_ after 50, 100, 250, 500, 1000 and 5000 ms (5-s depolarization from a HP of − 89 mV to *V*_max_). All values are presented as mean ± SEM (> 3 independent transfections). Statistics: unpaired Student’s *t* test of *r* values, *p < 0.05, **p < 0.01, ***p < 0.001 in comparison to respective WT. *n*, number of recordings; *WT*, wild-type

### Mutation S652L increases Ca^2+^-signaling during stimulation protocols simulating neuronal firing patterns

In order to predict the consequences of mutation S652L on Ca^2+^-influx during neuronal activity patterns, we simulated its activity during sustained upstate potentials and during action potential firing.

**Fig. 4:**
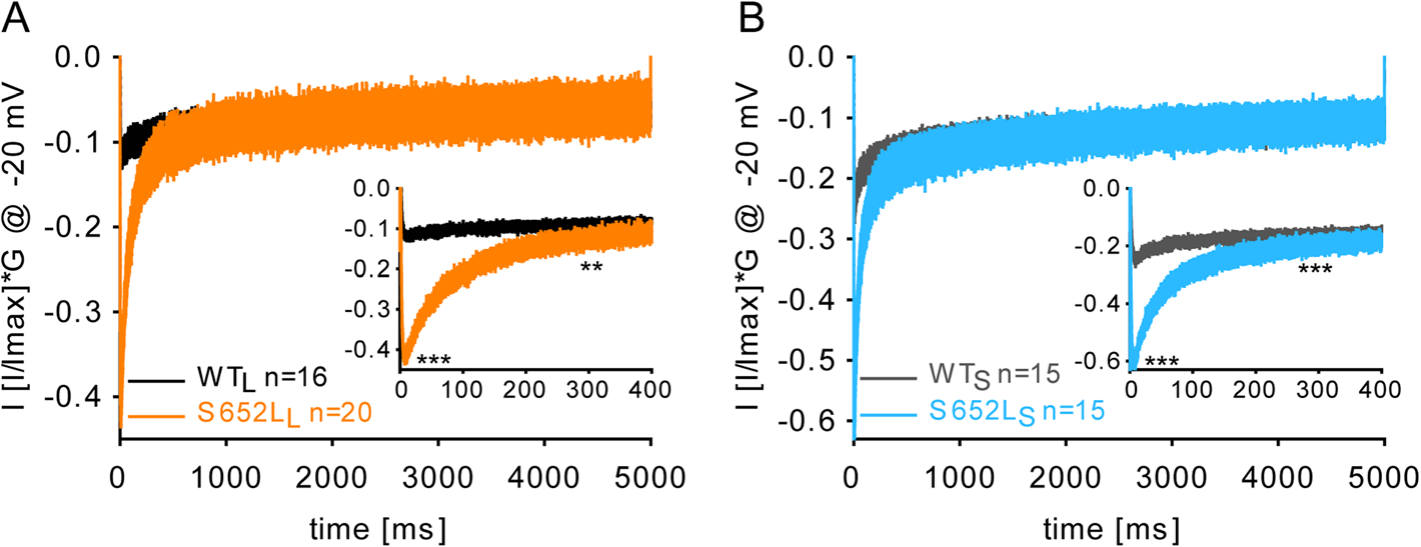
Mutation S652L increases Ca^2+^-influx during sustained upstate potentials. **a, b** Normalized representative *I*_Ca_ of WT_L_ vs S652L_L_ (A) and WT_S_ vs S652L_S_ (B) multiplied with the corresponding conductance at a physiologically relevant potential of -20 mV, which corresponds to ~ − 35-38 mV at physiological Ca^2+^-concentrations (WT_L_, 0.1253; S652L_L_, 0.4330; WT_S_, 0.2647; S652L_S_, 0.6325; Fig. 1) and plotted as a function of time. Insets show the first 400 ms. Data are presented as mean ± SEM for the *n*-numbers indicated. Statistics: unpaired Student’s *t* test of *I*_Ca_ at peak ([mean ± SEM; pA*pS]: WT_L_, − 0.12 ± 0.005, *n* = 16; S652L_L_, − 0.42 ± 0.005***, *n* = 20; WT_S_, − 0.24 ± 0.009, *n* = 15; S652L_S_, − 0.58 ± 0.011***, *n* = 15) and *I*_Ca_ after 300 ms ([mean ± SEM; pA*pS]: WT_L_, − 0.08 ± 0.006, *n* = 16; S652L_L_, − 0.13 ± 0.013**, *n* = 20; WT_S_, − 0.15 ± 0.008, *n* = 15; S652L_S_, − 0.19 ± 0.021, *n* = 15; **p < 0.01, ***p < 0.001). Data were collected from > 3 independent transfections

At low potentials, Cav1.3 channels can contribute to the formation of plateau potentials due to their known negative activation range (for a review, see [3]). This was described in medium spiny neurons when transient upstate potentials were induced by glutamatergic excitatory input [37]. To quantify changes of I_Ca_ amplitudes induced by the mutation at sustained subthreshold depolarizations we mimicked plateau potentials by prolonged depolarizations to − 20 mV with 15 mM Ca^2+^ as charge carrier. This voltage would correspond to ~− 35–38 mV at physiological Ca^2+^-concentrations [26] (Fig. 4a, b). The resulting *I*_Ca_ traces were normalized to maximal *I*_Ca_ at *V*_max_ in individual cells and corrected for splicing- and mutation-dependent differences in V_0.5_ by multiplying with the corresponding conductance measured for the different WT and mutant constructs at − 20 mV (derived from activation curves in Fig. 1c, d). As shown in Fig. 4a, b, mutated channels caused a marked and highly significant increase of *I*_Ca_ over the first 300 ms of the depolarization compatible with enhanced channel activity suitable to support upstate potentials during this time period (for details, see legend to Fig. 4).

**Fig. 5:**
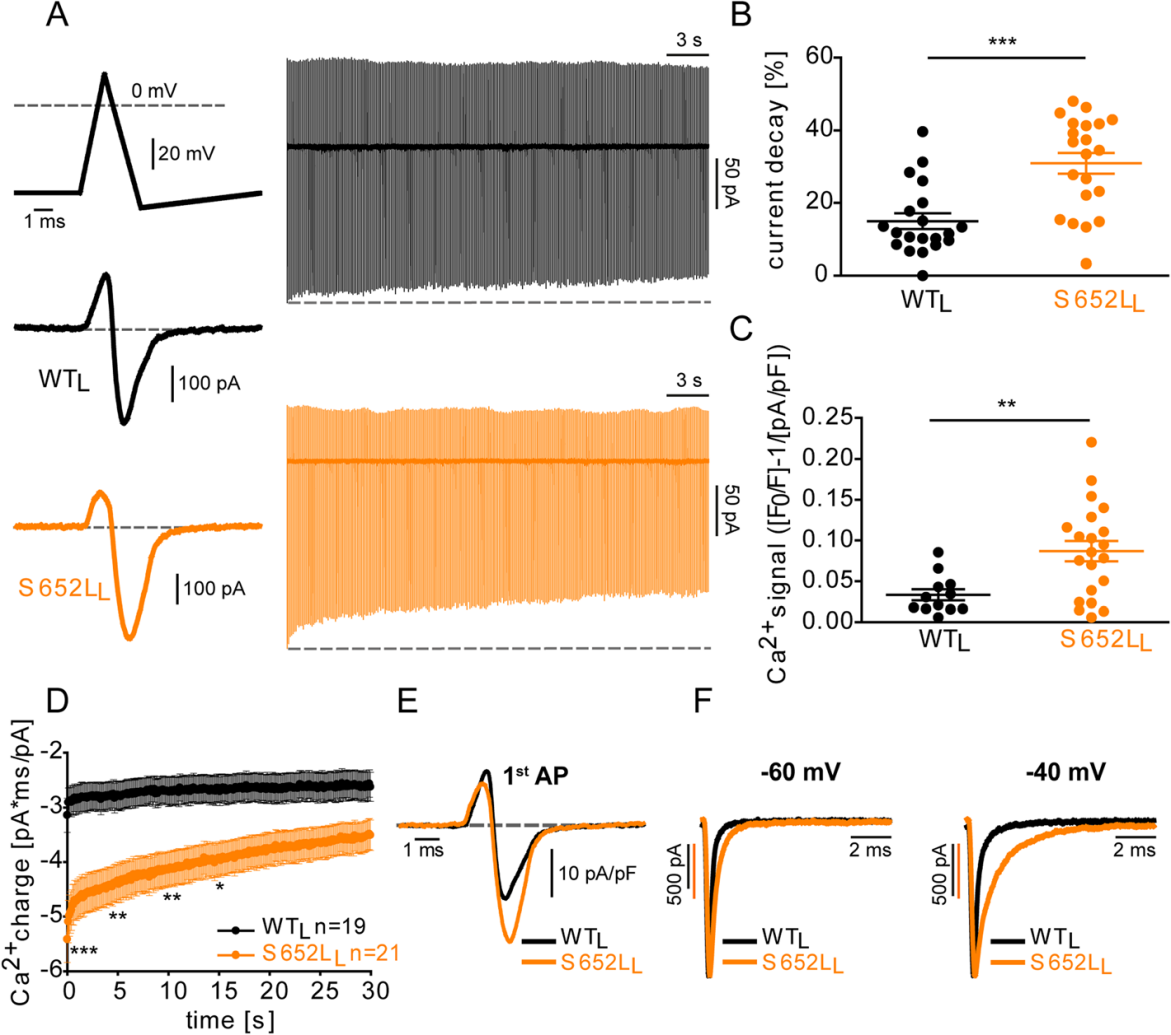
Mutation S652L increases intracellular Ca^2+^ during simulated action potential firing. **a** Upper left: Shape of single action potential waveform (APW) mimicked by the following voltage steps: HP: − 80 mV, − 80 to − 60 mV for 2.5 ms, − 60 to + 20 mV in 1 ms, + 20 to − 70 mV in 1.5 ms, − 70 to − 60 mV in 5 ms, − 60 mV for 90 ms. The corresponding *I*_Ca_ of WT_L_ and S652L_L_ are shown below. Right: Representative current responses of WT_L_ and S652L_L_ during 30 s of stimulation with APW-like stimuli at a frequency of 10 Hz. **b** Peak *I*_Ca_ of S652L_L_ Cav1.3 channels decayed faster than WT_L_ during stimulation. Statistics: unpaired student´s t-test ([mean ± SEM]; WT_L_, 14.94 ± 2.19, *n* = 20; S652L_L_, 30.94 ± 2.85***, *n* = 21; ***p < 0.001). **c** Average Ca^2+^-signal of WT_L_ and S652L_L_ expressing HEK-293 cells upon 30 s of stimulation. Ca^2+^-signal was normalized to baseline fluorescence (F_0_ [mean ± SEM]; WT_L_, 1.65 ± 0.20; S652L_L_, 1.38 ± 0.18) and current density (pA/pF [mean ± SEM]; WT_L_, − 11.93 ± 1.46; S652L_L_, − 8.51 ± 1.04) determined in a ramp protocol before the start of the train. S652L_L_ Cav1.3 channels showed higher levels of [Ca^2+^] than WT_L_ after 30 s of stimulation. Statistics: unpaired Student’s *t* test, **p < 0.01. **d** Ca^2+^-charge of WT_L_ and S652 L_L_ obtained by integrating the area of the I_Ca_ transient normalized to maximum I_Ca_ determined in a ramp protocol before the start of the train. Statistics: two-way ANOVA of selected time points (every 5 s), *p < 0.05, **p < 0.01, ***p < 0.001. **e** Overlay of 1^st^ AP of WT_L_ and S652L_L_ *I*_Ca_ transients normalized to current density (pA/pF) determined in a ramp protocol before the start of the train to demonstrate larger APs induced by mutation S652L. **f** Normalized representative *I*_Ca_ transients of WT_L_ and S652L_L_ obtained from repolarisations from + 80 mV to − 60 mV (left) or − 40 mV (right); scale bars correspond to the traces of the same color; for parameters and statistics see Table 3. The AP-like command voltage also triggered an outward current component occurring at the peak of the AP spike. We and others (see references in Ortner et al. [29]) have observed this previously. The outward component is likely composed of Q_ON_ and a passive non-LTCC component (also found in non-transfected cells, [29])

In order to predict the consequences of mutation S652L on Ca^2+^-influx during action potential firing we simultaneously measured *I*_Ca_ and cytosolic Ca^2+^-responses upon stimulation of HEK-293 cells transfected with WT_L_ and S652L_L_ with 10-Hz-trains of action potential-like waveforms (APW) (Fig. 5a; for details, see legend) [26, 38]. These stimuli resulted in typical *I*_Ca_ transients (Fig. 5a) with maximal *I*_Ca_ reached during the repolarization phase of the APW [29, 38, 39]. Peak *I*_Ca_ slowly decreased during trains, an effect that was significantly enhanced by the mutation (Fig. 5a, b). However, the mutation enhanced the increase in intracellular Ca^2+^-levels measured simultaneously during this stimulation protocol (Fig. 5c). To explain this discrepancy, we measured the total Ca^2+^-charge during the 30-s train. This was significantly higher in S652L mutated channels even after 15 s of stimulation (Fig. 5d; for details, see legend). It was not due to higher S652L channel expression, because we normalized data to current density for each cell. As shown in Fig. 5e, *I*_Ca_ amplitude during APW repolarization was significantly larger for S652L as compared to WT_L_ (peak 1^st^ AP [pA/pF]: WT_L_, − 12.43 ± 1.95, *n* = 19; S652L_L_, − 33.64 ± 3.13***, *n* = 21; unpaired Student’s *t* test, ***p < 0.001). This could be explained by the more negative activation voltage range as well as a pronounced slowing of *I*_Ca_ deactivation. Deactivation of tail currents following repolarizations from + 80 mV to − 60 mV or − 40 mV was significantly slower in S652L_L_ as compared to WT_L_. This was primarily caused by a decrease in the contribution of the fast component and an increase of the slow component of the bi-exponential deactivation process (Fig. 5f; for statistics, see Table 3). Thus, higher Ca^2+^-levels and Ca^2+^-charge can be attributed to the slower deactivation kinetics and higher current amplitudes induced by mutation S652L during action potential like firing.

**Table 3:** Tail current parameters of mutation S652L

α_1_-subunit	τ_fast_ (ms)	τ_slow_ (ms)	C (pA/pF)	A_fast_ (pA/pF)	A_slow_ (pA/pF)	Half width (ms)	Norm. tail area (pA*ms)	n
− 60 mV								
WT_L_	0.16 ± 0.01	0.91 ± 0.08	− 0.003 ± 0.001	− 0.79 ± 0.02	− 0.12 ± 0.02	0.31 ± 0.02	− 0.44 ± 0.02	14
S652L_L_	0.20 ± 0.01*	0.81 ± 0.04	− 0.007 ± 0.001**	− 0.51 ± 0.02***	− 0.37 ± 0.03***	0.41 ± 0.02***	− 0.65 ± 0.03***	16
− 40 mV								
WT_L_	0.19 ± 0.02	1.12 ± 0.07	− 0.006 ± 0.001	− 0.65 ± 0.02	− 0.24 ± 0.01	0.37 ± 0.02	− 0.65 ± 0.04	14
S652L_L_	0.39 ± 0.03***	1.95 ± 0.08***	− 0.047 ± 0.003***	− 0.29 ± 0.02***	− 0.54 ± 0.02***	0.92 ± 0.07***	− 1.87 ± 0.09***	16

Normalized tail currents were fitted to a bi-exponential decay (*τ*_fast,_ *τ*_slow:_ time constants of slow and fast component; *A*_fast_, *A*_slow_: amplitudes of slow and fast components; C: non-inactivating component). Statistics: Mann-Whitney *U* test, *p < 0.05, **p < 0.01, ***p < 0.001. All values are presented as mean ± SEM (> 3 independent transfections). *n*, number of recordings

### Mutation S652W produces a loss of Cav1.3 channel function

**Fig. 6:**
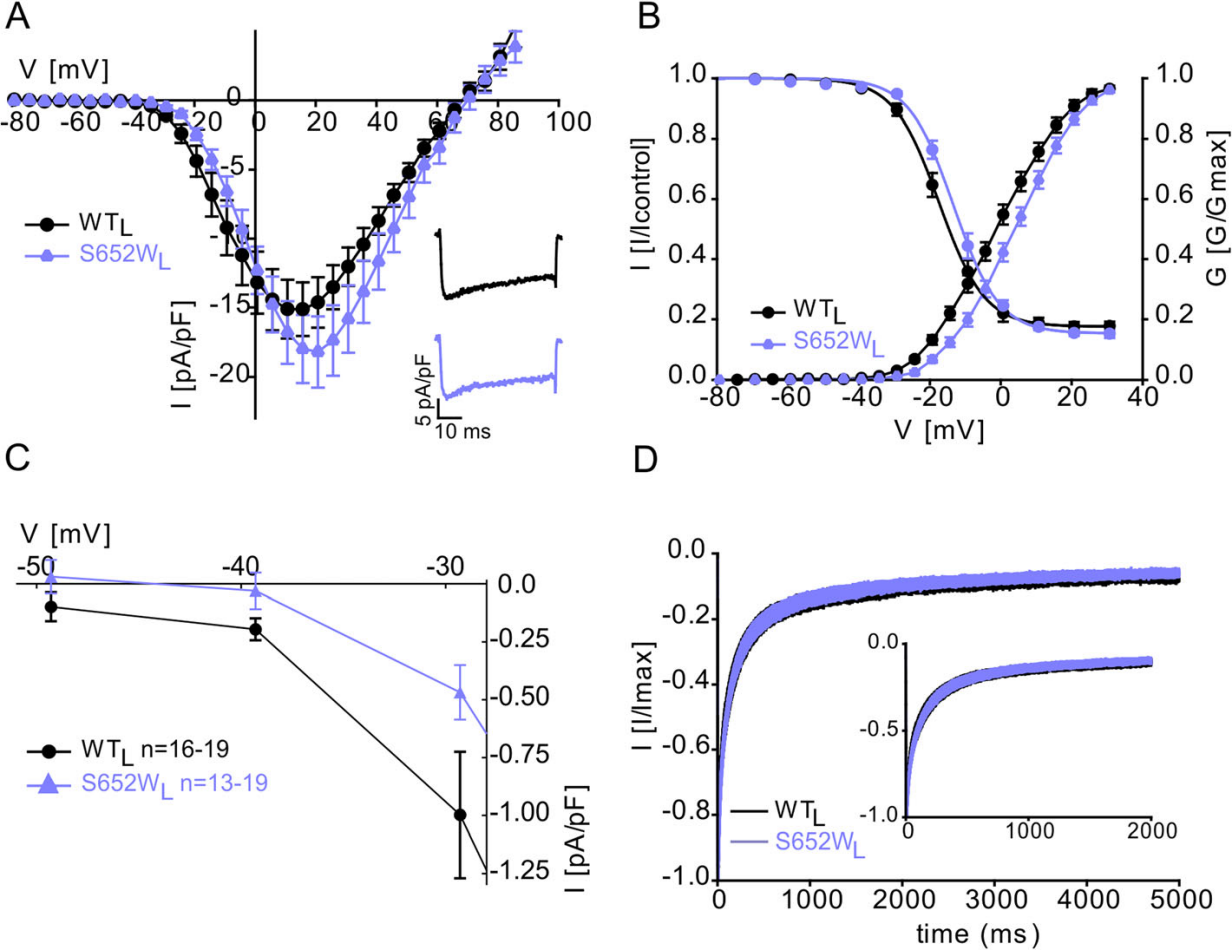
Mutation S652W induces gating changes compatible with a loss-of-function phenotype. **a** Current-voltage relationship (*I*_Ca_; mean ± SEM) of WT_L_ vs S652W_L_ recorded in parallel on the same day as described in Fig. 1. Inset: Representative *I*_Ca_ traces of WT_L_ and S652W_L_ upon depolarization to the *V*_max_. **b** Normalized steady-state activation and inactivation curves of WT_L_ vs S652W_L_. Data are presented as mean ± SEM; for parameters and statistics see Table 4. **c** Window currents of WT_L_ vs S652W_L_ were calculated as in Fig. 1e, f. Data are presented as means for the indicated number of experiments. **d** Inactivation kinetics of WT_L_ vs. S652W_L_ during a 5-s depolarization to *V*_max_ with Ca^2+^ as a charge carrier showing no difference in inactivation kinetics between WT_L_ and S652W_L_. Inset shows the first 2000 ms. Data are presented as mean ± SEM; for statistics and number of experiments, see Table 5. The significant shift of activation and inactivation half-maximal voltage to positive voltages and the absence of a change in inactivation time course was independently confirmed in an independent set of experiments using HEK-293 cells stably expressing β_3_ and α2δ-1 subunits with essentially identical results. Statistics: one-way ANOVA followed by Bonferroni post hoc test, *p < 0.05. Data are represented as mean ± SEM for the *n*-numbers indicated. Data were collected from > 3 independent transfections

Our data predict that only *CACNA1D* mutations that are able to enhance Cav1.3 channel activity can confer high risk for neurodevelopmental symptoms. Accordingly, like any of the other previously described pathogenic variants, S652L has not been reported in the genomes of 141,456 control individuals free from pediatric disease (gnomAD database [21];). In contrast, the pathogenic potential should be low or absent from missense mutations causing gating defects favoring reduced function, as outlined above, in mice [40, 41] and humans [24, 25]. Interestingly, the gnomAD database also reports the rare variant p.Ser672Trp (chr3: 53757881 C>G, human reference genome hg19) variant (S652W), located in the same position as S652L, in three healthy unrelated individuals. PolyPhen2 and SIFT predict a probably damaging (score: 1.00) and deleterious (100%) effect on protein function, respectively, but these algorithms cannot predict gating changes. This provided us with a unique opportunity to further test our above hypothesis by studying the biophysical properties of S652W. As shown in Fig. 6b, this mutation has opposite effects on the voltage-dependence of gating compared to S652L. Both steady-state activation and inactivation were significantly shifted to more positive voltages by 4–5 mV (for statistics see Table 4).

**Table 4:** Activation and inactivation parameters of mutation S652W

	Activation				Inactivation			
α_1_-subunit	V_0.5_ (mV)	k (mV)	V_rev_ (mV)	n	V_0.5,inact_ (mV)	k (mV)	Non-inactivating (%)	n
WT_L_	− 0.50 ± 1.48	9.32 ± 0.26	67.1 ± 1.60	19	− 17.5 ± 1.32	5.94 ± 0.28	17.4 ± 1.45	16
S652 W_L_	4.23 ± 1.27*	8.73 ± 0.22	64.6 ± 2.12	19	− 13.1 ± 1.22*	5.73 ± 0.30	15.1 ± 1.06	13

Parameters were obtained by fitting normalized activation curves (*G*/*G*_max_) or inactivation curves (*I*/*I*_control_) as in Table 1. All values are presented as mean ± SEM (> 3 independent transfections). Statistics: unpaired Student’s *t* test, *p < 0.05 compared to WT_L_. *n*, number of recordings. *V*_0.5_, half maximal activation/inactivation voltage; *V*_rev_, reversal potential; *WT*, wild- type

Consequently, window currents were not increased and even tended to be shifted to more positive voltages also compatible with a loss-of-function at threshold voltages (Fig. 6c). S652W also failed to enhance channel function by other mechanisms: it did neither slow inactivation kinetics (Fig. 6d; for statistics, see Table 5) nor change the fraction of non-inactivating current as evident from the steady-state inactivation analysis (Fig. 6b).

**Table 5:** Normalized inactivation kinetic parameters of mutation S652W

	Remaining I_Ca_ [%]						
α_1_-subunit	*r*_50_	*r*_100_	*r*_250_	*r*_500_	*r*_1000_	*r*_5000_	*n*
WT_L_	60.45 ± 4.22	47.88 ± 4.09	30.88 ± 3.21	20.44 ± 2.26	14.61 ± 1.85	7.59 ± 0.86	11
S652W_L_	62.86 ± 2.34	49.49 ± 2.35	31.35 ± 2.13	20.61 ± 1.99	13.85 ± 1.53	6.35 ± 0.74	13

*r* values represent the fraction of remaining *I*_Ca_ after 50, 100, 250, 500, 1000, and 5000 ms upon a 5-s depolarization to the voltage of maximal inward current (*V*_max_). All values are presented as meanM (> 3 independent transfections). No significant differences were found by unpaired Student’s *t* test of *r* values, compared to WT_L_. *n*, number of recordings; *WT*, wild-type

### Molecular modeling of Cav1.3 WT, S652L, and S652W α_1_-subunits

**Fig. 7:**
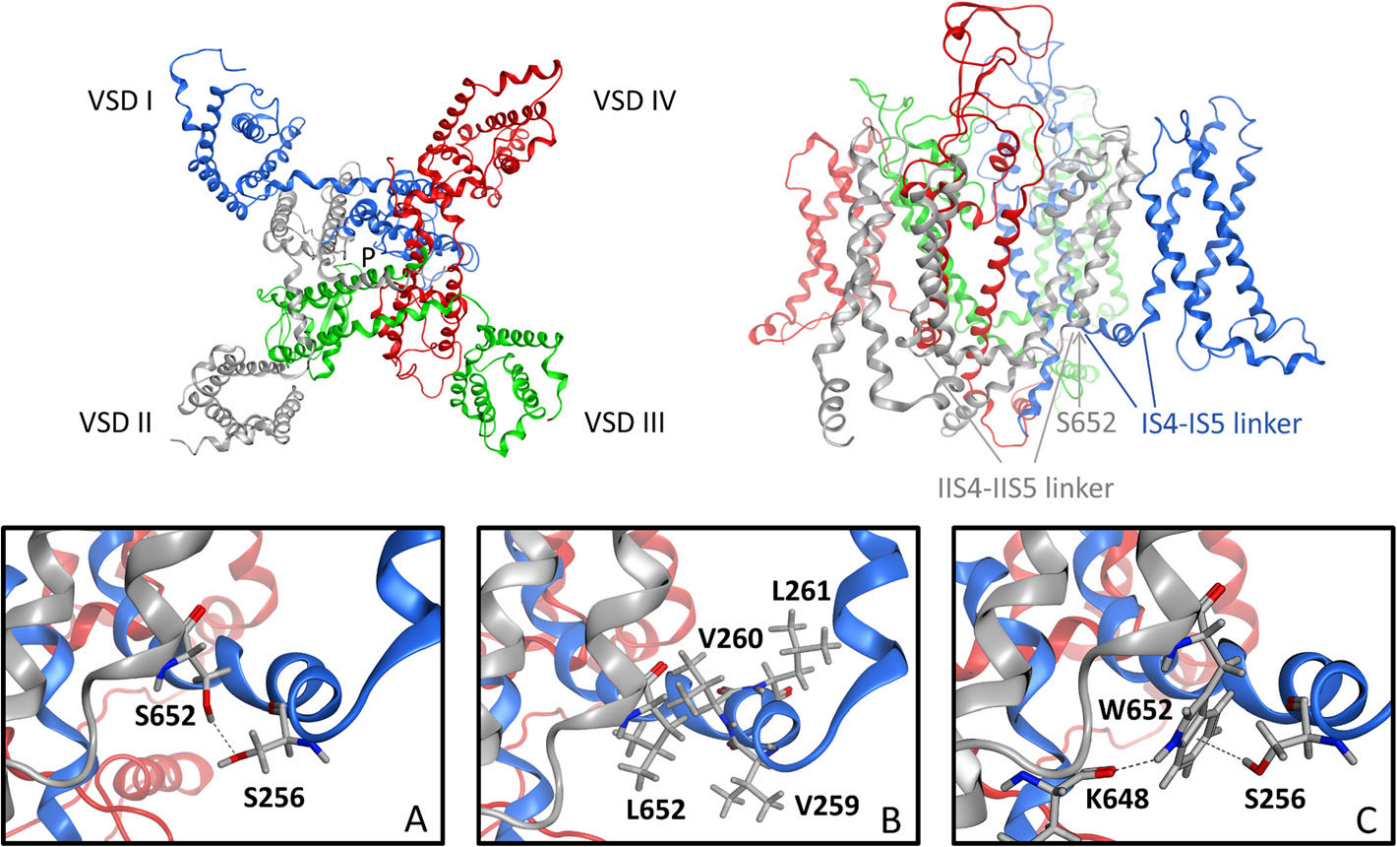
Molecular modeling of Cav1.3 WT α_1_-subunits, mutations S652L and S652W. Top: Top view and side view of the Cav1.3 α_1_-subunit structure. The region involving the inter-domain interactions (IIS4-S5–IS4-IS5) affected by the mutation is highlighted (left). Bottom: **a** WT inter-domain interaction of S652 in repeat II and S256 in the S4-S5 linker in repeat I. **b** Weaker hydrophobic interactions of the mutated residue L652 with the hydrophobic cloud in the S4-S5 linker of repeat I. **c** Stabilizing effect of the W652 mutation; the tryptophan residue can form an intra-domain hydrogen bond with the backbone of K648 and due to its aromatic character an inter-domain pi-H interaction with S256.

On the structural level, the loss of a newly discovered inter-domain hydrogen bond connecting the S4–S5 linkers of repeats II and I could explain the gating differences between the two variants (Fig. 7). Our Cav1.3 channel homology model, based on the cryo-EM structure of the Cav1.1 α_1_-subunit [30], localizes S652 at the C-terminal end of the S4–S5 linker in channel repeat II (Fig. 7, upper, left). The S4–S5 linkers in each repeat are known to form contacts with the cytoplasmic end of their corresponding S6 helices, which together form the inner mouth of the channel (activation gate [30];). This allows the S4–S5 linkers to transmit voltage-sensor movements to the activation gate. Our model predicts that S652 forms a hydrogen bond with S256 in the S4–S5 linkers of the neighboring repeat I (Fig. 7a). Therefore, this hydrogen bond connects the S4–S5 linkers in two different repeats and could be crucial for controlling the voltage-sensitivity of channel gating. This is supported by our finding that in the S652L mutant, this inter-domain interaction is not stabilized by any hydrogen bonds. Instead, leucine forms much weaker hydrophobic contacts with the residues V259, V260, and L261 located in the S4–S5 linkers of repeat I (Fig. 7b). In contrast, the aromatic side chain of the tryptophan in the S652 W mutant is capable of forming an inter-domain pi-H interaction with S256 in IS4–S5 as well as an intra-domain hydrogen bond with its own IIS4-S5 backbone (K648) (Fig. 7c), which should also allow a rigidifying effect similar as in the WT channel.

Together with our functional data, this reveals the importance of an inter-domain hydrogen bond for normal electromechanical coupling in Cav1.3 channels, which has not been described before. Weakening this interaction by substituting S652 with leucine causes a dramatic change in channel gating. Since we modeled the mutation with the activation gate in a closed channel conformation, this hydrogen bond likely stabilizes the channel in a closed state. Its weakening would favor the transition to and/or the stabilization in the open state, which can explain the strong shift of the voltage-dependence of activation to more negative potentials. Stabilization of the open state can also explain the slower transition from the open to the closed state evident as slowing of deactivation in the S652L mutant channel at a given voltage.

### Mutation S652L increases the sensitivity of Cav1.3 channels for inhibition by the dihydropyridine LTCC blocker isradipine

The fact that pathogenicity is associated with enhanced channel function but that reduced Cav1.3 channel function in the brain is not associated with CNS symptoms (see above), make available LTCC blockers a potential therapeutic option for the symptomatic treatment of individuals affected by gain-of-function mutations.

**Fig. 8:**
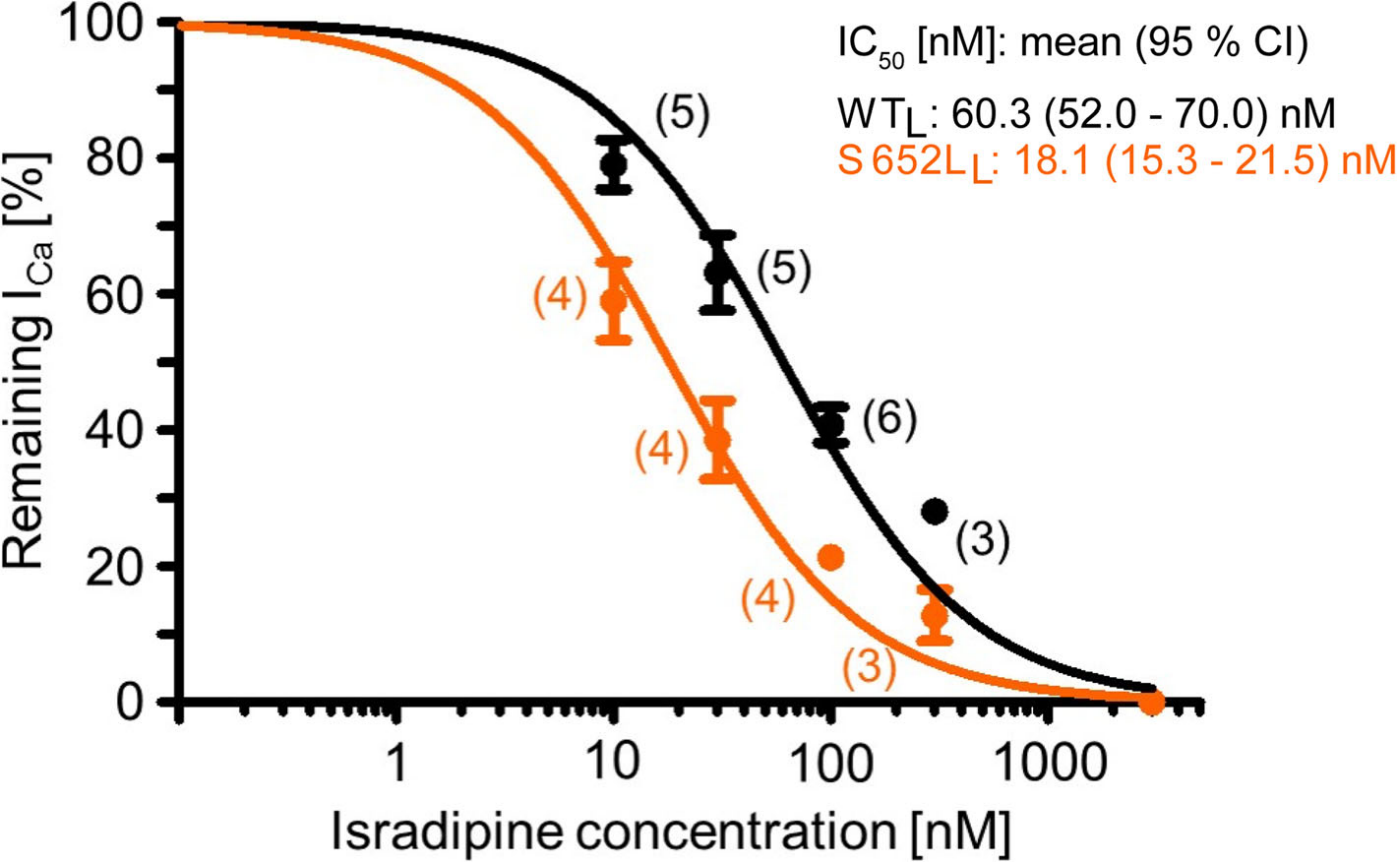
Mutation S652L shows higher isradipine sensitivity. Concentration–response curves for WT_L_ and S652L_L_ steady-state *I*_Ca_ inhibition by isradipine. Data are presented as mean ± SEM for the *n*-numbers indicated. Curves were fitted using a Hill slope = 1 and top-bottom fixed (bottom = 0; top = 100). IC_50_ values are given as means with 95% confidence interval. The statistical significance was determined using the extra sum-of-squares *F* test (*p* < 0.0001).

These drugs, such as the dihydropyridines (DHPs) nifedipine, felodipine, or isradipine, are safely used since decades for the treatment of arterial hypertension and angina. Since DHPs preferentially bind to channels in an inactivated state [42, 43], mutations affecting the gating properties, such as S652L may change the sensitivity of the channel for inhibition by DHPs. In order to quantify mutation-induced changes on DHP sensitivity, we assessed inhibition of the C-terminally long WT and S652L mutant channels by the DHP isradipine using a standard square pulse protocol (100 ms to *V*_max_, 0.1 Hz, HP: − 89 mV). As illustrated in Fig. 8, S652L-mutated channels required significantly lower isradipine concentrations for channel inhibition with a 3–4-fold decrease of their half maximal inhibitory concentration (IC_50_, mean (95% coincidence interval); WT_L_: 60.3 (52.0 – 70.0) nM, S652L_L_: 18.1 (15.3 – 21.5) nM; Fig. 8). This finding suggests that DHPs with good brain penetrance, such as isradipine [29, 44] may preferentially inhibit S652L-mutated Cav1.3 channels. Based on their good clinical safety profile this encourages therapeutic trials with DHPs in the affected individuals.

## Discussion

In this study, we provide compelling evidence for the *CACNA1D* S652L variant as a high-risk and likely disease-causing mutation in two individuals of the Deciphering Developmental Disorders cohort (decipher.sanger.ac.uk) of children with severe, undiagnosed developmental disorders [23]. This evidence builds on a detailed biophysical characterization that demonstrates gating changes able to also induce enhanced channel activity as is typical for six other de novo germline missense mutations in patients with ASD with and without other neurodevelopmental symptoms [14–18, 20]. Our data adds the *CACNA1D* gene to the other 12 developmental-disorder-linked genes identified in the Deciphering Developmental Disorders study and thus further increases its diagnostic yield. Moreover, since S652L has also been found in APAs as a somatic mutation we also confirm S652L as a disease-causing mutation in patients with primary aldosteronism [33]. We also demonstrate that although heterozygous missense mutations in the same position can be damaging, they can have opposite effects on channel function and, in the case of Cav1.3 α_1_-subunits, may comprise low or no pathogenic risk when the resulting gating changes does not support a gain-of-function. This complicates predictions of the disease-causing role of rare de novo *CACNA1D* variants in genetic studies and emphasizes the need for functional analysis as described in this report. We clearly show this for S652W. Whereas neither S652L nor one of the other pathogenic *CACNA1D* mutations is reported in the gnomAD database, the heterozygous S652W variant is reported in three neurologically apparently healthy individuals in this database [21]. In contrast to S652L, this variant tended to shift the window current to more positive rather than negative voltages, which is due to the positive shift of the voltage-dependence of activation and inactivation. This nicely fits our prediction that only *CACNA1D* mutations which can also support channel gain-of-function confer high risk for neurodevelopmental disorders. It is also in line with previous studies in mice (for a review, see [3]) and humans [24, 25]. These found that heterozygous loss-of-function of Cav1.3 is unlikely to cause symptomatic neurodevelopmental disorders and even homozygous loss of Cav1.3 function has not been reported to cause neuropsychiatric behavioral changes [3, 24, 25, 41]. Therefore, a unifying feature of all pathogenic mutations described so far is the potential to induce gating changes that can enhance Cav1.3 function during neuronal activity. The potential for enhanced channel function may, however, vary between different neurons. For example, in neurons firing from more depolarized membrane potentials, the negative shift in steady-state inactivation may also reduce the availability of Cav1.3 channels.

S652L adds to another six de novo missense mutations functionally characterized so far by us [14–16] and others [17, 18, 20, 38] in a total of seven patients with neurodevelopmental disorders. Functionally they fall into two major classes. Channel gain-of-function is either predominantly induced by stabilizing a large non-inactivating current component (type 1: in particular, G403D, G407R) or by inducing a strong shift of activation voltage to more negative potentials (type 2: A749G, I750M and V401L) [14–17], as also observed for S652L. Both types of mutations enhance intracellular Ca^2+^-load when expressed in HEK-293 (A749G, [38]; S652L, this paper) or GLT muscle cells (G407R, [14]). Pronounced negative shifts of activation and/or pronounced slowing of channel inactivation can therefore be taken as "diagnostic" feature for the pathogenicity of *CACNA1D* de novo mutations in patients with neurodevelopmental disease.

Importantly, our data also strongly suggest that pathogenicity can also be assumed if the same variant has also been reported as a somatic mutation in APAs. As shown here, this is the case for S652L [33] and likewise for G403D, I750M and V401L [17, 45].

We have also observed a small but significant shift of *V*_rev_ (Fig. 1, Table 1) to more negative voltages. This could indicate a potential change in ion selectivity by the mutation. Interestingly, we have also detected similar shifts by other Cav1.3 α_1_-subunit gain-of-function mutations [16]. If confirmed by single-channel recordings, this may also contribute to the mutation-induced pathological signaling changes.

Very recently identified de novo mutation V259A has been reported in another severely affected individual, a 1-year-old male with seizures, global developmental delay and primary aldosteronism [19]. So far this mutation has not been functionally characterized. Although its pathogenic potential appears to be high based on two different APA mutations reported in the same position (V259D, V259G) [46]), final proof requires functional analysis as described here.

Finally, another important and clinically highly relevant result of our study was the observation that Cav1.3 channels harboring the S652L-mutation require lower concentrations of the DHP isradipine for inhibition. This can be explained by the known voltage-dependence of DHP action due to their preferred binding to inactivated channel states [29, 42]. S652L induces a pronounced negative shift of the voltage-dependence of inactivation thus increasing the availability of inactivated channels. This preclinical finding is a strong motivation to test if repurposing of already available DHPs could ameliorate symptoms in affected individuals. Since also other mutations inducing strong shifts in steady-state inactivation are likely to increase DHP sensitivity (e.g. A749G, [14]), this treatment approach, if successful, could also be offered to individuals with other *CACNA1D* mutations.

## Limitations

Although our data strongly support *CACNA1D* as a high-risk gene for neurodevelopmental disorders and emphasize the need of functional analysis to distinguish likely pathogenic (able to increase Cav1.3 activity) from non-pathogenic de novo mutations (unable to increase Cav1.3 activity), our studies do not provide insight into altered signaling cascades downstream of Cav1.3 channels. This will require introduction of one or more of these human mutations into the mouse *Cacna1d* gene for electrophysiological and biochemical studies in native cells. Such animal models will also allow to address the important question, if currently available LTCC blocker, such as isradipine, felodipine of nimodipine, can normalize cellular function and, perhaps, even behavioral phenotypes in these mice. Although LTCC blockers may normalize the mutation-induced increase in channel function after diagnosis, it is possible that the mutation may have already caused permanent developmental deficiencies resistant to drug treatment. Therefore the clinical potential of this therapy needs to be tested in small clinical trials in affected individuals.

## Conclusions

Taken together our data have important implications for genetic diagnostics. We provide evidence that *CACNA1D* is a neurodevelopmental disorder-linked gene. Although initially considered to cause high risk only for ASD with or without intellectual disability [14], the increasing number of affected individuals, including S652L, now strongly indicates that the majority presents with a more severe phenotype. This can involve seizures, intellectual disability and, due to the role of Cav1.3 for aldosterone and insulin secretion [40, 46]), also with (often transient, [17, 18]) endocrine symptoms. Our findings with S652W (the loss-of-function mutation) emphasize that, in the case of *CACNA1D*, the amino acid position itself does not allow predictions about the disease risk of a variant, even if bioinformatics prediction tools provide high scores for protein damage. A high probability for pathogenicity can also be assumed if a variant identical to the germline mutation has also been found in at least two different individuals as a somatic mutation in an APA or an aldosterone-producing cell-cluster [34, 47, 48]. Our report should raise awareness for the pathogenic potential of *CACNA1D* mutations, especially in patients without additional congenital endocrine symptoms as diagnostic features. At present, de novo *CACNA1D* missense mutations may be underdiagnosed in clinical practice.

## Supplementary information

**Additional file 1: Figure S1.** Expression of WT and S652L Cav1.3 α_1_-subunits in HEK-293 cells. (A) Expression of C-terminally long and short WT and S652L α_1_-subunits by Western blot analysis. One representative Western blot of >3 experiments from >2 membrane preparations of transfected HEK-293 cells stably expressing β_3_ and α_2_δ-1 is shown. The apparent molecular mass of the full length forms of the long and short Cav1.3 α_1_-subunit splice variants obtained under our experimental conditions is indicated. (B) Quantification of relative total protein expression levels was carried out by integrating densities of WT and mutant signals and normalization to the loading control α-tubulin (α-tub). Statistics: unpaired student´s t-test compared to WT (S652 L_L_: 118.26 ± 23.43, *n* = 4; S652 L_S_: 129.62 ± 8.94, *n* = 3). Data are presented as mean ± SEM.

**Additional file 2: Figure S2.** Relationship of Q_ON_-gating charge movement and integrated tail current amplitude (I_tail_) as an indirect estimate of open probability. (A) Channel open probability was estimated from the slope of the Q_ON_-I_tail_ relationships measured at V_rev_. Slopes were obtained by linear regression: I_tail_/Q_ON_ [mean ± SEM; ms^-1^]: WT_L_: -7.22 ± 0.916, r^2^ = 0.72, *n* = 26; S652L_L_: -4.24 ± 0.657, r^2^ = 0.72, *n* = 25; slopes are significantly different: *F* = 6.43, *p* = 0.015, *F* test). (B) Representative traces obtained by depolarization to the reversal potential from a HP of -89 mV. Data were collected from more than three independent transfections.

## Data Availability

The datasets generated and/or analyzed during the current study are available from the corresponding author on reasonable request.

## Abbreviations

APA: Aldosterone-producing adenoma
APW: Action potential-like waveform
ASD: Autism spectrum disorder
Cav: Voltage-gated Ca^2+^-channel
CDI: Ca^2+^-dependent inactivation
CNS: Central nervous system
DHP: Dihydropyridine
*G*: Conductance
HEK: Human embryonic kidney
HP: Holding potential
*I*_Ba_: Inward Ba^2+^-currents
*I*_Ca_: Inward Ca^2+^-currents
*P*_O_: Open probability
*Q*_ON_: “ON” gating charge
*V*_0.5_: Half-maximal activation voltage
VDI: Voltage-dependent inactivation
VGCC: voltage-gated Ca^2+^-channel
*V*_max_: Potential of maximal inward current
*V*_rev_: Reversal potential
WT: Wild-type
