# Supplementary figures and images for: Biophysical classification of a CACNA1D de novo mutation as a high-risk mutation for a severe neurodevelopmental disorder
Source: Mol Autism. 2020 Jan 8;11:4. doi: 10.1186/s13229-019-0310-4 (PMC6950833; doi:10.1186/s13229-019-0310-4)

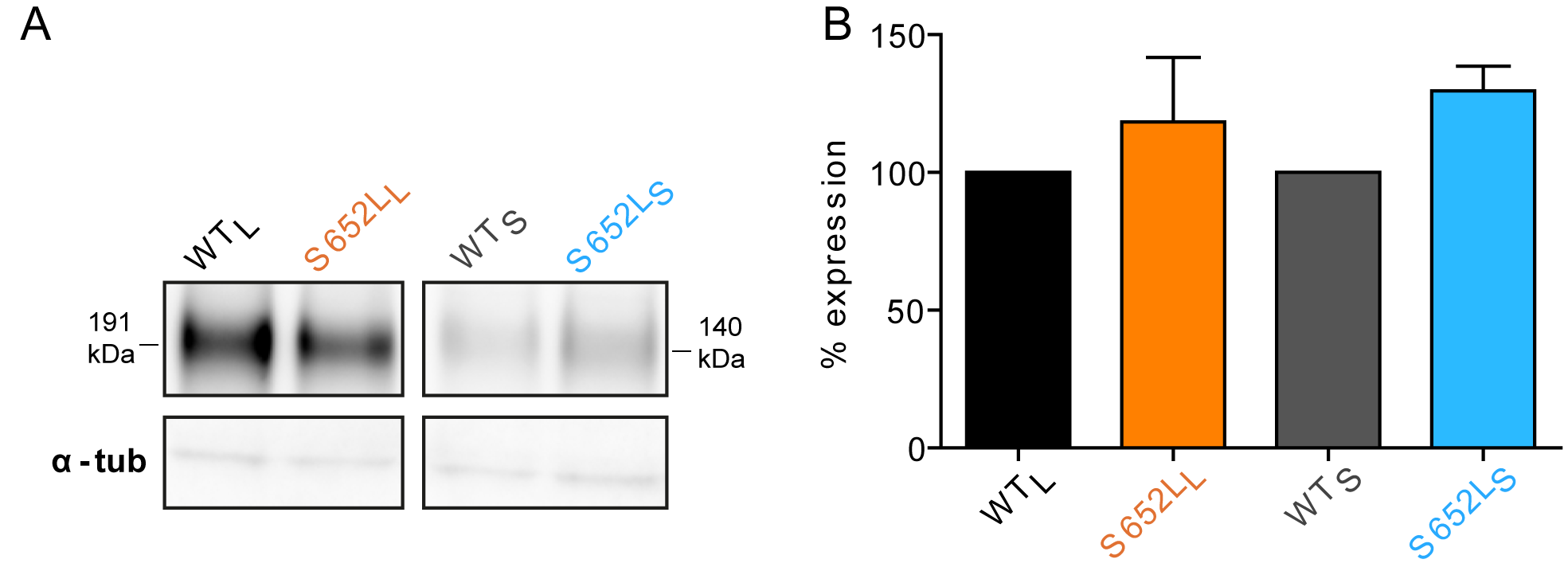

Supplement: Supplementary file 1 — Additional file 1: Figure S1. Expression of WT and S652L Cav1.3 α1-subunits in HEK-293 cells. (A) Expression of C-terminally long and short WT and S652L α1-subunits by Western blot analysis. One representative Western blot of >3 experiments from >2 membrane preparations of transfected HEK-293 cells stably expressing β3 and α2δ-1 is shown. The apparent molecular mass of the full length forms of the long and short Cav1.3 α1-subunit splice variants obtained under our experimental conditions is indicated. (B) Quantification of relative total protein expression levels was carried out by integrating densities of WT and mutant signals and normalization to the loading control α-tubulin (α-tub). Statistics: unpaired student´s t-test compared to WT (S652 LL: 118.26 ± 23.43, n = 4; S652 LS: 129.62 ± 8.94, n = 3). Data are presented as mean ± SEM. [file 13229_2019_310_MOESM1_ESM.tif]

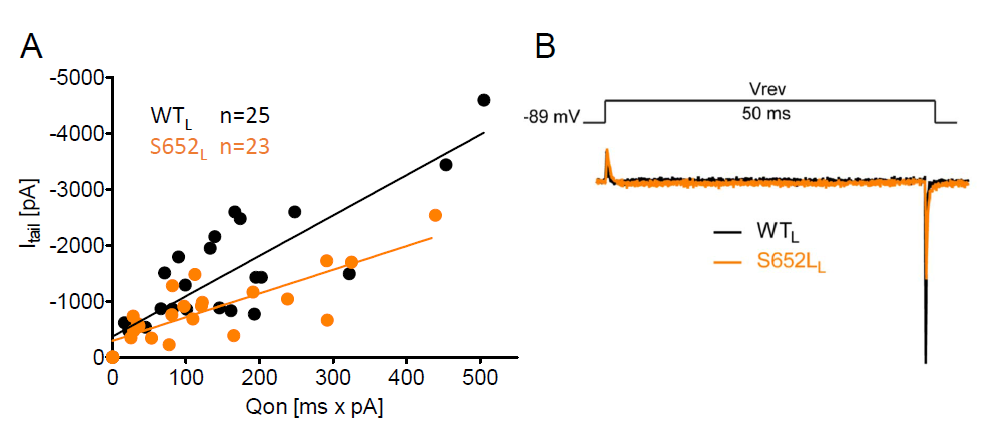

Supplement: Supplementary file 2 — Additional file 2: Figure S2. Relationship of QON-gating charge movement and integrated tail current amplitude (Itail) as an indirect estimate of open probability. (A) Channel open probability was estimated from the slope of the QON-Itail relationships measured at Vrev. Slopes were obtained by linear regression: Itail/QON [mean ± SEM; ms-1]: WTL: -7.22 ± 0.916, r2 = 0.72, n = 26; S652LL: -4.24 ± 0.657, r2 = 0.72, n = 25; slopes are significantly different: F = 6.43, p = 0.015, F test). (B) Representative traces obtained by depolarization to the reversal potential from a HP of -89 mV. Data were collected from more than three independent transfections. [file 13229_2019_310_MOESM2_ESM.tif]
